# Embryonic hindbrain patterning genes delineate distinct cardio-respiratory and metabolic homeostatic populations in the adult

**DOI:** 10.1038/s41598-017-08810-4

**Published:** 2017-08-22

**Authors:** Jenny J. Sun, Teng-Wei Huang, Jeffrey L. Neul, Russell S. Ray

**Affiliations:** 1 Department of Neuroscience, Baylor College of Medicine, Houston, Texas USA; 2 Verna and Marrs McLean Department of Biochemistry and Molecular Biology, Baylor College of Medicine, Houston, Texas USA; 3 Department of Molecular Physiology and Biophysics, Baylor College of Medicine, Houston, Texas USA; 4 Program in Developmental Biology, Baylor College of Medicine, Houston, Texas USA; 5 Department of Pediatrics, Baylor College of Medicine, Houston, Texas USA; 6 Center for Cell and Gene Therapy, Baylor College of Medicine, Houston, Texas USA; 7 Jan and Dan Duncan Neurological Research Institute, Texas Children’s Hospital, Houston, Texas USA; 8 McNair Medical Institute, TX-77030, Houston, USA

## Abstract

Previous studies based on mouse genetic mutations suggest that proper partitioning of the hindbrain into transient, genetically-defined segments called rhombomeres is required for normal respiratory development and function in neonates. Less clear is what role these genes and the neurons they define play in adult respiratory circuit organization. Several Cre drivers are used to access and study developmental rhombomeric domains (*Eng1* ^*Cre*^, *HoxA2-Cre*, *Egr2* ^*Cre*^, *HoxB1* ^*Cre*^, *and HoxA4-Cre*) in the adult. However, these drivers show cumulative activity beyond the brainstem while being used in intersectional genetic experiments to map central respiratory circuitry. We crossed these drivers to conditional DREADD mouse lines to further characterize the functional contributions of Cre defined populations. In the adult, we show that acute DREADD inhibition of targeted populations results in a variety of not only respiratory phenotypes but also metabolic and temperature changes that likely play a significant role in the observed respiratory alterations. DREADD mediated excitation of targeted domains all resulted in death, with unique differences in the patterns of cardio-respiratory failure. These data add to a growing body of work aimed at understanding the role of early embryonic patterning genes in organizing adult respiratory homeostatic networks that may be perturbed in congenital pathophysiologies.

## Introduction

The perpetual rhythm of breathing is essential for survival and emerges through the interactions of a highly redundant and anatomically complex array of brainstem neuronal networks. Disruptions to these networks are believed to play a role in several congenital diseases with respiratory deficits including Rett Syndrome^1–4^, Congenital Central Hypoventilation Syndrome (CCHS)^5–7^, and Sudden Infant Death Syndrome (SIDS)^8–10^ as well as adult homeostatic pathophysiologies such as Sudden Unexpected Death in Epilepsy (SUDEP)^11^. The brainstem homeostatic circuitry is functionally patterned by a combination of developmental molecular and genetic events as well as activity dependent cues such as the transition from an amniotic environment to air breathing and suckling^12–16^. Thus, a necessary step toward diagnosis and treatment of respiratory dysfunction is to determine the relative role of developmental and environmental influences that result in the mature configuration of the adult respiratory neural network.

Genetically defined hindbrain rhombomere (r) segments are a primary scaffold in the patterning of the hindbrain neuro-epithelium and proper partitioning is required for normal respiratory development during embryogenesis and immediately after birth. While it is often presumed that rhombomeric patterning is important for the function of the adult respiratory network, there is little data that has directly examined this question in adults, as direct perturbation of hindbrain development often results in neonate lethality, developmental compensatory events, and cell-non-autonomous effects including perturbation of neighboring rhombomeric domains (even if anatomically intact)^17–27^.

**Figure 1:**
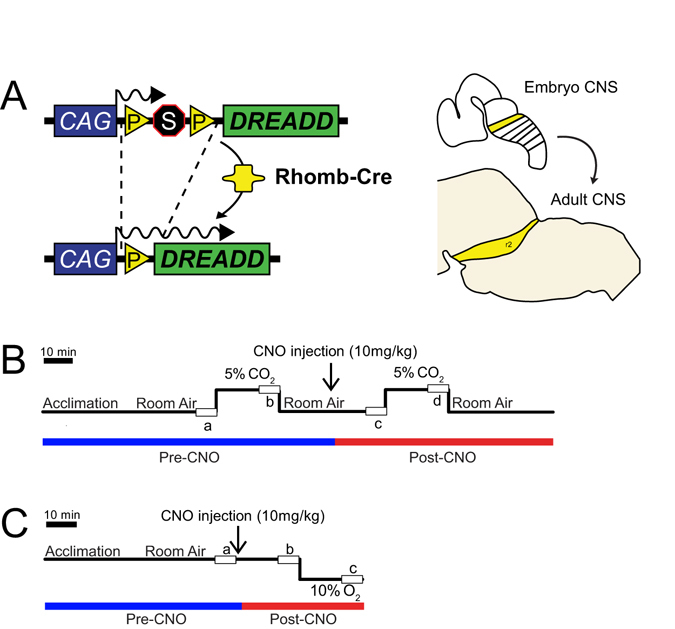
Breeding schematic and respiratory protocols. (**A**) The *RC::P_hM4D* allele is combined with 5 different *rhombomere-specific-Cre* lines to achieve cell specific expression of the hM4D receptor in embryonically-defined domains in the adult. Shown as an example is rhombomere 2 (*HoxA2-Cre)*. (**B**) Hypercapnic respiratory protocol. The animal is placed into the respiratory chamber and allowed to acclimate under baseline room air conditions. The animal is then exposed to 20 minutes of 5% CO_2_ followed by 20 minutes of room air. The animal is then injected intraperitoneally with 10 mg/kg of clozapine-N-oxide (CNO) followed by another 20 minutes of post-CNO room air, 20 minutes of 5% CO_2_, and 20 minutes of room air. (**C**) Hypoxic respiratory protocol. This protocol is similar to the hypercapnic protocol except that no pre-CNO 10% O_2_ measurement is taken to avoid potential confounds of hypoxic plasticity on the post-hypoxic response.

To examine how neural populations that are captured by the expression of early embryonic patterning genes relevant to rhombomeric development become integrated into adult respiratory homeostatic networks, we utilized a suite of widely used mouse lines for marking and functionally studying rhombomeric domains that cumulatively span the majority of the mouse brainstem (*Eng1* ^*Cre*^ for r1^28^, *HoxA2-Cre* for r2^29^, *Egr2* ^*Cre*^ for r3&5^30^, *HoxB1* ^*Cre*^ for r4^31^, and *HoxA4-Cre* for r7&8^32^) but also show activity in areas of the mid and forebrain as well as the spinal cord. These lines were paired with a Cre responsive inhibitory hM4Di DREADD mouse line^33^ (*RC::P_hM4Di)* and a newly derived, Cre responsive excitatory hM3Dq *(RR2P)* mouse line (Fig. 1A). The combination of these DREADD^34^ alleles with the five Cre drivers enables us to bypass embryonic lethality while avoiding developmental mispatterning and compensatory events to functionally assess embryonic rhombomere demarcated adult brainstem and other populations in a cell autonomous fashion. In combination with whole-body plethysmography while measuring oxygen consumption to address possible perturbations to metabolic regulation, we are able to accurately measure multiple respiratory and temperature parameters under room air, hypercapnic, and hypoxic conditions (Fig. 1B,C) to assess the contributions of neurons captured by early embryonic patterning genes important in rhombomeric patterning in the organization of the adult brainstem homeostatic network.

Cumulatively, our work supports a role for the persistent contribution of embryonic patterning in the adult respiratory network despite postnatal inputs that significantly refine these networks. Our work also offers several points of consideration for studies using these Cre drivers in mapping brainstem respiratory circuits using DREADDS and other effector molecules for acute manipulation. Our anatomical studies show that many of these commonly used Cre drivers have activity outside of the brainstem that must be taken into account if these drivers are used in single or dual recombinase mapping approaches^26, 27, 33, 35^. Our respiratory studies demonstrate that acute inhibition of *Eng1* ^*Cre*^, *HoxA2-Cre*, *Egr2* ^*Cre*^, *HoxB1* ^*Cre*^, and *HoxA4-Cre* derived populations results in a variety of distinct phenotypic changes in respiratory rate (V_f_), tidal volume (V_T_), minute ventilation $({\dot{{\rm{V}}}}_{{\rm{E}}})$, oxygen consumption $({\dot{{\rm{V}}}}_{{\rm{O2}}})$, ${\dot{{\rm{V}}}}_{{\rm{E}}}$/${\dot{{\rm{V}}}}_{{\rm{O2}}}$ and waveform patterns under three tested respiratory conditions chosen for their relevance to congenital pathophysiologies. Notably, by measuring oxygen consumption, we observed that some respiratory changes were accompanied by matched changes in ${\dot{{\rm{V}}}}_{{\rm{O2}}}$, while others showed a disproportionate relationship between changes in breathing and metabolism leading to a hyper- or hypoventilatory baseline state, suggesting that some baseline respiratory and chemosensory phenotypes may be an indirect consequence of changes to metabolic regulation. These findings indicate that ${\dot{{\rm{V}}}}_{{\rm{E}}}$/${\dot{{\rm{V}}}}_{{\rm{O2}}}$ measurements are required to draw firm conclusions from similar prior^26, 27, 33, 35^ and future studies using DREADDs to map respiratory circuits. DREADD mediated excitation of targeted rhombomeric domains all resulted in death, with unique differences in the patterns of cardio-respiratory failure, which may also shed insight on possible mechanisms in SUDEP.

Our work adds to the sparse studies of adult neural populations defined by the expression of early embryonic patterning genes important in rhombomeric development in the functional organization of the adult brainstem cardio-respiratory and metabolic networks. Our work further highlights several technical and physiological considerations that must be taken into account for future acute perturbation studies to map specific neural circuits involved in respiratory homeostasis and metabolic control that will yield important clues about congenital pathophysiologies such as SIDS, CCHS, and Rett Syndrome as well as seizure associated autonomic vulnerabilities.

## Results

### *Eng1*^*Cre*^, *HoxA2-Cre*, *Egr2*^*Cre*^, *HoxB1*^*Cre*^, and *HoxA4-Cre* drivers mark rhombomeric domains in the embryo and the adult as well as domains outside of the brainstem

**Figure 2:**
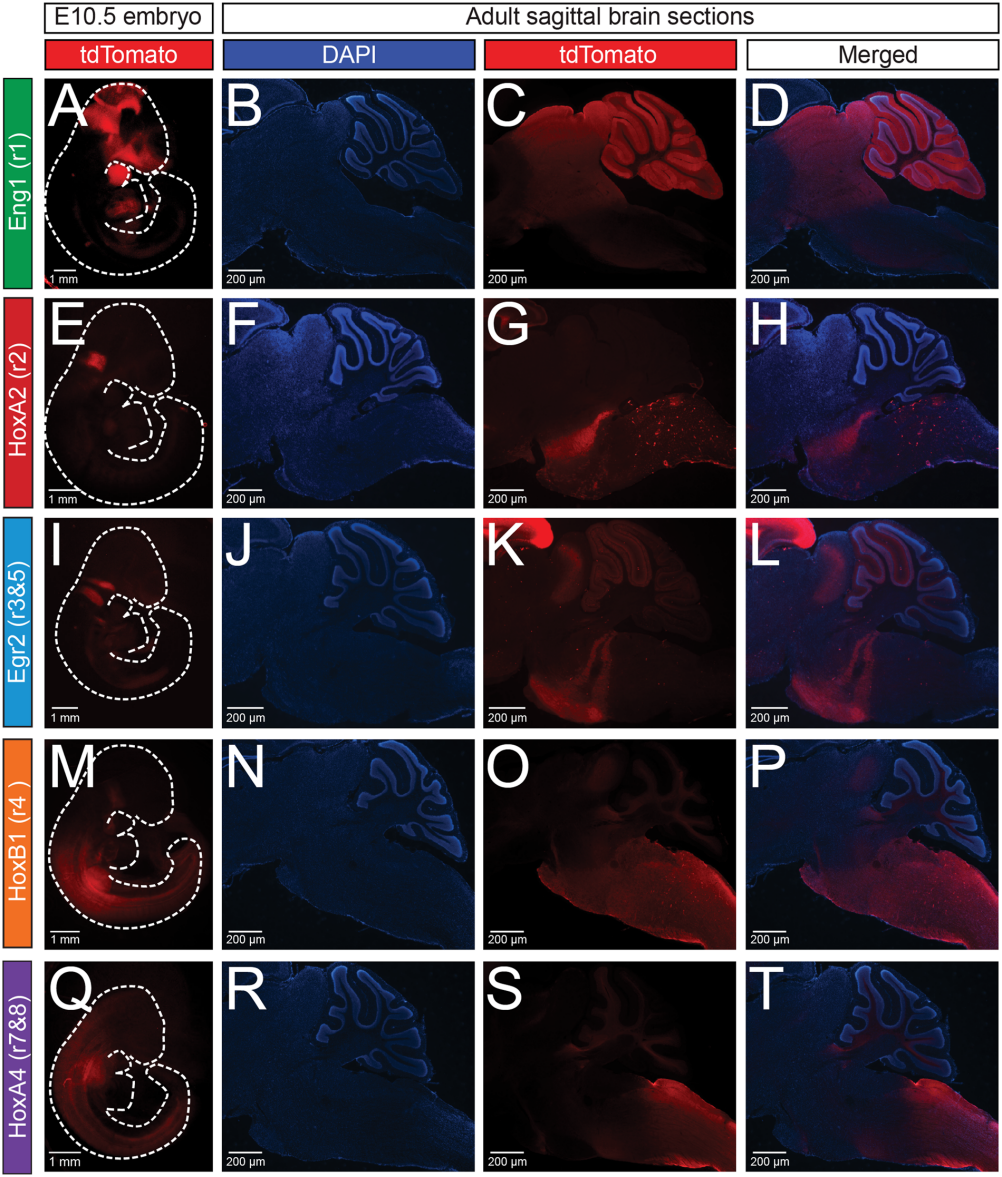
The *Eng1* ^*Cre*^, *HoxA2-Cre*, *Egr2* ^*Cre*^, *HoxB1* ^*Cre*^, and *HoxA4-Cre* drivers capture broad rhombomeric domains in the embryo and adult. The five *rhombomere-specific-Cre* lines were crossed with *Ai9* mice to express tdTomato in a Cre-specific manner. In column 1, *rhombomere-Cre; Ai9* E10.5 whole embryos were cleared using hydrogel cross-linking followed by lipid extraction by SDS treatment. In columns 2–4, DAPI and tdTomato fluorescence of sagittal brainstem cryosections (30 μm) from adult *rhombomere-Cre; Ai9* animals are shown. Embryo and adult expression can be seen in the *Eng1* ^*Cre*^ **(A–D)**, *HoxA2-Cre* (**E–H)**, *Egr2* ^*Cre*^ **(I–L)**, *HoxB1* ^*Cre*^ **(M–P)**, and *HoxA4-Cre* **(Q–T**) domains.

To examine the domains captured by and to assess the recombination efficiency of each of the *Cre* drivers, we crossed *Eng1* ^*Cre*^ *, HoxA2-Cre, Egr2* ^*Cre*^ *, HoxB1* ^*Cre*^ *, and HoxA4-Cre* animals to the *Ai9* line that expresses a floxed tdTomato (Fig. 2). We examined tdTomato expression in both E10.5 whole embryos and adult sagittal brainstem sections, where we observed distinct patterns that confirm the expression patterns seen in previous studies in which the activity of *Cre* expression has been well documented. More detailed descriptions are provided in the next section. As the *Ai9* line is a *Rosa26* knock-in reporter allele configured similarly to the *RC::P_hM4D* and *RR2P* lines, we expect hM4D and hM3D expression to recapitulate the patterns seen in our genetic fate mapping.

### hM4D DREADD mediated acute perturbation of rhombomeric domains results in a variety of respiratory, metabolic and temperature phenotypes

Breathing is exquisitely tuned to maintain physiological homeostasis, and adapts accordingly to various environmental and physiological challenges. Both high CO_2_ and low O_2_ are powerful respiratory stimuli, as the changes in ${{\rm{P}}}_{{{\rm{CO}}}_{2}}$ and ${{\rm{P}}}_{{{\rm{O}}}_{2}}$ in the blood lead to increased ventilation, and are thought to involve the activity of multiple types of brainstem neurons. To test the role of embryonic rhombomeric domains under baseline conditions as well as high CO_2_ (hypercapnic) and low O_2_ (hypoxic) conditions, we crossed *Eng1* ^*Cre*^, *HoxA2-Cre*, *Egr2* ^*Cre*^, *HoxB1* ^*Cre*^, and *HoxA4-Cre* animals to the *RC::P_hM4D* mouse line for acute perturbation in the adult under either a hypercapnic protocol or hypoxic protocol (Fig. 1), with room air and temperature measurements included in both. For room air and hypercapnic conditions, we measured ventilatory parameters both before and after CNO administration to experimental animals and sibling controls. For hypoxic conditions, we only measured ventilatory parameters after CNO administration to avoid the potential confounds of hypoxic plasticity and the post-hypoxic response. We examined the respiratory rate (V_f_), tidal volume (V_T_), minute ventilation (${\dot{{\rm{V}}}}_{{\rm{E}}}$, V_f_ × V_T_), oxygen consumption (${\dot{{\rm{V}}}}_{{{\rm{O}}}_{2}}$), and minute ventilation normalized to oxygen consumption (${\dot{{\rm{V}}}}_{{\rm{E}}}$/${\dot{{\rm{V}}}}_{{{\rm{O}}}_{2}}$). We also examined potential changes in pattern by examining the frequency of apneas and sighs and the coefficients of variation for interbreath interval (IBI) and amplitude as indications of periodic and volume instability. Finally, temperature measurements were performed at the beginning of the assay (pre-CNO), in the middle of the assay (pre-CNO), immediately at the end of the assay (post-CNO), and 30 minutes after the end of the assay at room temperature (post-CNO).

#### Acute perturbation of *Eng1*^*Cre*^ neurons in adult mice results in increased oxygen consumption and stability under room air conditions, and reduced hypercapnic and hypoxic ventilatory responses

The *Eng1* ^*Cre*^ domain spans rhombomere 1 of the hindbrain as well as virtually all cells in the midbrain^28^ and includes several populations of cells previously implicated in respiratory physiology: the majority of the noradrenergic locus coeruleus^36^ as well as a subset of dopaminergic and serotonergic neurons^37^.

**Figure 3:**
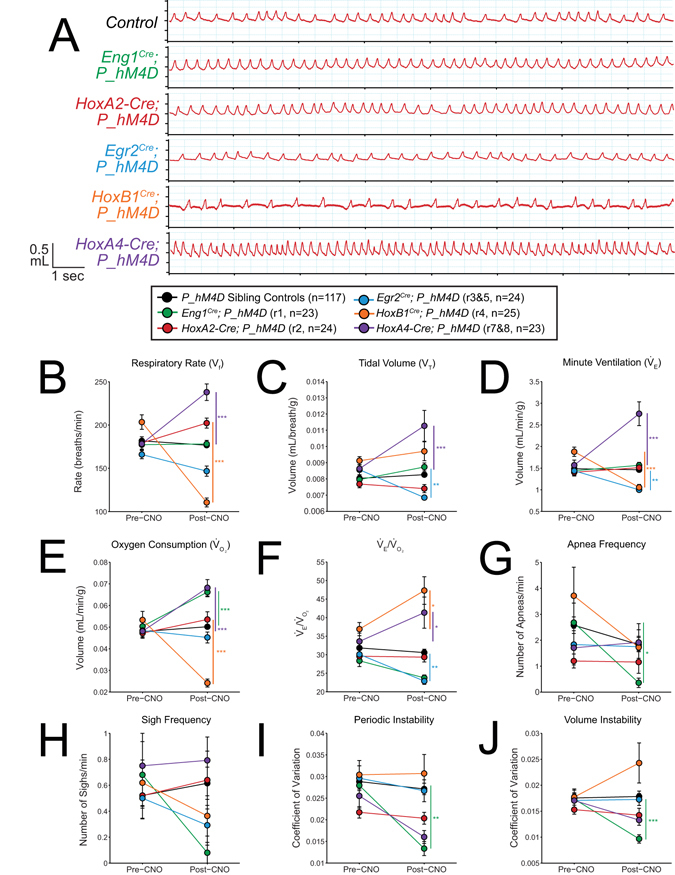
CNO-hM4D perturbation of rhombomeric domains in the adult animal results in a variety of respiratory phenotypes under room air conditions. (**A**) Representative traces of respiratory wavesforms in *P_hM4D* sibling controls and *rhombomere-Cre*; *P_hM4D* mice after CNO injection under room air conditions. (**B–F**) Quantification of pre- and post-CNO respiratory parameters. Perturbation of rhombomere-derived populations under room air conditions results in increases, decreases, and no change in respiratory rate (**B**), tidal volume (**C**), minute ventilation (**D**), oxygen consumption (**E**), ${\dot{{\rm{V}}}}_{{\rm{E}}}$/${\dot{{\rm{V}}}}_{{{\rm{O}}}_{2}}$ (**F**), apnea frequency (**G**), sigh frequency (**H**), periodic instability (**I**), and volume instability (**J**) as compared to pre-CNO values and sibling controls. Statistical significance was determined using a linear mixed-effects regression model with animal type (experimental or control) and CNO treatment (pre or post) as fixed effects and animal ID as a random effect. *p < 0.05, **p < 0.01, ***p < 0.001.

**Figure 4:**
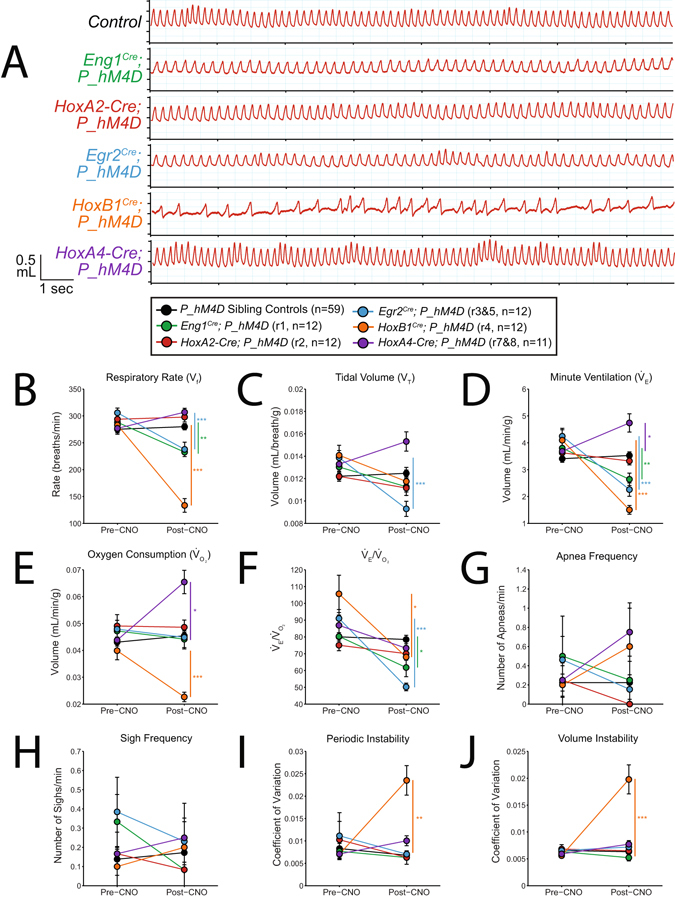
CNO-hM4D perturbation of rhombomeric domains in the adult animal results in a variety of respiratory phenotypes under hypercapnic (5% CO_2_) conditions. (**A**) Representative traces of respiratory wavesforms in *P_hM4D* sibling controls and *rhombomere-Cre; P_hM4D* mice after CNO injection under hypercapnic conditions. (**B–F**) Quantification of pre- and post-CNO respiratory parameters. Perturbation of rhombomere-derived populations under room air conditions results in increases, decreases, and no change in respiratory rate (**B**), tidal volume (**C**), minute ventilation (**D**), oxygen consumption (**E**), ${\dot{{\rm{V}}}}_{{\rm{E}}}$/${\dot{{\rm{V}}}}_{{\rm{O2}}}$ (F), apnea frequency (**G**), sigh frequency (**H**), periodic instability (**I**), and volume instability (**J**) as compared to pre-CNO values and sibling controls. Statistical significance was determined using a linear mixed-effects regression model with animal type (experimental or control) and CNO treatment (pre or post) as fixed effects and animal ID as a random effect. *p < 0.05, **p < 0.01, ***p < 0.001.

**Figure 5:**
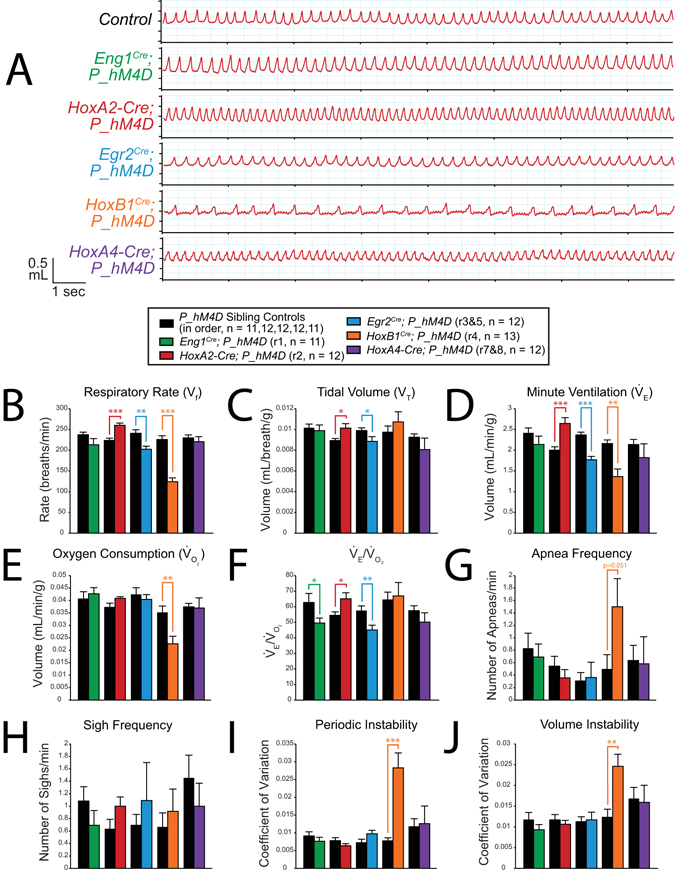
CNO-hM4D perturbation of rhombomeric domains in the adult animal results in a variety of respiratory phenotypes under hypoxic (10% O_2_) conditions. (**A**) Representative traces of respiratory wavesforms in *P_hM4D* sibling controls and *rhombomere-Cre; P_hM4D* mice after CNO injection under hypoxic conditions. (**B–F**) Quantification of pre- and post-CNO respiratory parameters. Perturbation of rhombomere-derived populations under hypoxic conditions results in increases, decreases, and no change in respiratory rate (**B**), tidal volume (**C**), minute ventilation (**D**), oxygen consumption (**E**), ${\dot{{\rm{V}}}}_{{\rm{E}}}$/${\dot{{\rm{V}}}}_{{{\rm{O}}}_{2}}$ (**F**), apnea frequency (**G**), sigh frequency (**H**), periodic instability (**I**), and volume instability (**J**) as compared to sibling controls. Statistical significance was determined using a linear mixed-effects regression model with animal type (experimental or control) as a fixed effect. *p < 0.05, **p < 0.01, ***p < 0.001.

**Figure 6:**
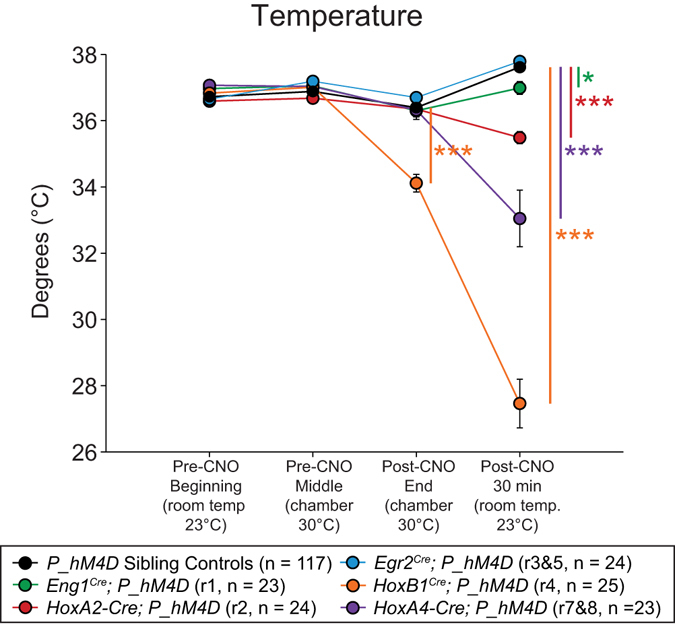
CNO-hM4D perturbation of rhombomeric domains in the adult animal results in significant reductions in body temperature. Temperature was taken at the start of respiratory assays (Pre-CNO Beginning), immediately before CNO injection (Pre-CNO Middle), at the end of respiratory assays (Post-CNO End), and 30 minutes at room temperature after the end of the assay (Post-CNO 30 min). Statistical significance was determined using a linear mixed-effects regression model with animal type (experimental or control) as a fixed effect. *p < 0.05, **p < 0.01, ***p < 0.001.

Under room air conditions, after CNO administration, *Eng1* ^*Cre*^ *; P_hM4D* animals showed an increase in ${\dot{{\rm{V}}}}_{{\rm{O2}}}$ (+0.016 mL/min/g, p < 0.0001, Fig. 3E, Supplemental Figs S1D and S2D) and decreases in apnea number (−2.32 apneas/min, p = 0.040, Fig. 3G, Supplemental Figs S1F and S2F), periodic instability (−0.015 in CV, p = 0.0065, Fig. 3I, Supplemental Figs S1H and S2H), and volume instability (−0.0076 in CV, p < 0.0001, Fig. 3J, Supplemental Figs S1I and S2I) as compared to pre-CNO values and sibling controls. Under hypercapnic conditions, *Eng1* ^*Cre*^; *P_hM4D* animals showed reductions in V_f_ (−55.541 breaths/min, p = 0.0018, Fig. 4B, Supplemental Fig. S1A), $\dot{{\rm{V}}}$_E_ (−1.1602 mL/min/g, p = 0.0070, Fig. 4D, Supplemental Fig. S1C), and $\dot{{\rm{V}}}$_E_/${\dot{{\rm{V}}}}_{{{\rm{O}}}_{2}}$ (−18.5339, p = 0.040, Fig. 4F, Supplemental Fig. S1E). Six males and six females were assayed under the hypercapnic assay and no significant differences pre- or post-CNO were seen in respiratory or metabolic parameters under room air or hypercapnic conditions. Male *Eng1* ^*Cre*^; *P_hM4D* mice showed a lower post-CNO end temperature than females (36.58 vs. 35.17 °C, p = 0.020) but no difference was seen 30 minutes after the end of the assay. Under hypoxic conditions, minor but statistically insignificant changes in individual respiratory parameters resulted in a small deficit in $\dot{{\rm{V}}}$_E_/${\dot{{\rm{V}}}}_{{{\rm{O}}}_{2}}$ (−13.2007, p = 0.049, Fig. 5F, Supplemental Fig. S2E) as compared to sibling controls. Eight females and three males were assayed under the hypoxic assay and female *Eng1* ^*Cre*^; *P_hM4D* animals showed a higher $\dot{{\rm{V}}}$_E_ as compared to male animals under hypoxic conditions (2.39 vs. 1.46 mL/min/g, p = 0.026) with no other significant differences seen under room air or hypoxic conditions. Overall, *Eng1* ^*Cre*^; *P_hM4D* animals also showed a small reduction in temperature at 30 minutes after the end of the assay as compared to sibling controls (−0.5039 °C, p = 0.025, Fig. 6, Supplemental Figs S1J and S2J).

#### Acute perturbation of *HoxA2-Cre* neurons in adult mice results in an enhanced hypoxic ventilatory response and a temperature deficit 30 minutes after the end of respiratory assays

As previously described, the *HoxA2-Cre* domain captures cells from rhombomere 2, which can be seen as a distinct stripe in the embryo and the adult (Fig. 2E–H), and includes several populations of cells previously implicated in respiratory physiology: a subset of the medullary raphé nucleus^26^, and a very small subset of the locus coeruleus and A7 nuclei^36^.

No change was seen under room air or hypercapnic conditions after CNO administration (Figs 3 and 4, Supplemental Figs S3 and S4). Three females and nine males were assayed under the hypercapnic protocol. Interestingly, while the grouped cohort showed no overall change, under hypercapnic conditions females showed minimal change or an increase while males showed a decrease in V_T_ (+0.0005 vs. −0.0016 mL/breath/g, p = 0.018) and ${\dot{{\rm{V}}}}_{{\rm{E}}}$/${\dot{{\rm{V}}}}_{{{\rm{O}}}_{2}}$ (+11.91 vs. −10.53, p = 0.021). Under hypoxic conditions after CNO administration, *HoxA2-Cre*; *P_hM4D* animals showed an enhanced V_f_ (+36.54 breaths/min, p < 0.0001, Fig. 5B, Supplemental Fig. S4A), V_T_ (+0.0012 mL/breath/g, p = 0.013, Fig. 5C, Supplemental Fig. S4B), ${\dot{{\rm{V}}}}_{{\rm{E}}}$ (+0.6435 mL/min/g, p = 0.00043, Fig. 5D, Supplemental Fig. S4C), and ${\dot{{\rm{V}}}}_{{\rm{E}}}$/${\dot{{\rm{V}}}}_{{{\rm{O}}}_{2}}$ (+10.6826, p = 0.021, Fig. 5F, Supplemental Fig. S4E) as compared to sibling controls. Nine males and three females were assayed under the hypoxic protocol and no significant differences were seen in respiratory or metabolic parameters under room air or hypoxic conditions. Overall, *HoxA2-Cre*; *P_hM4D* animals showed a reduced temperature at 30 minutes after the end of both assays as compared to sibling controls (−2.0375 °C, p < 0.0001, Fig. 6, Supplemental Figs S3J and S4J).

#### Acute perturbation of *Egr2*^*Cre*^ neurons in adult mice results in reduced room air, hypercapnic, and hypoxic ventilation

As previously described, the *Egr2* ^*Cre*^ domain captures cells derived from rhombomeres 3 and 5, which can be seen as two distinct stripes in the embryo and the adult (Fig. 2I–L) and include several populations of cells that have been previously implicated in respiratory physiology: a subset of serotonergic neurons that contribute to the full hypercapnic ventilatory response in minute ventilation^26^, a majority of Phox2b retrotrapezoid nucleus (RTN) neurons, and a small subset of A5 noradrenergic neurons^27, 38^.

Under room air conditions, after CNO administration, *Egr2* ^*Cre*^; *P_hM4D* animals showed reduced V_T_ (−0.0017 mL/breath/g, p = 0.0016, Fig. 3C, Supplemental Figs S5B and S6B), ${\dot{{\rm{V}}}}_{{\rm{E}}}$ (−0.4391 mL/min/g, p = 0.0043, Fig. 3D, Supplemental Figs S5C and S6C), and ${\dot{{\rm{V}}}}_{{\rm{E}}}$/${\dot{{\rm{V}}}}_{{{\rm{O}}}_{2}}$ (−7.1889, p = 0.0030, Fig. 3F, Supplemental Figs S5E and S6E) as compared to pre-CNO values and sibling controls. Under hypercapnic conditions, *Egr2* ^*Cre*^; *P_hM4D* animals showed blunted V_f_ (−68.046 breaths/min, p < 0.0001, Fig. 4B, Supplemental Fig. S5A), V_T_ (−0.0046 mL/breath/g, p < 0.0001, Fig. 4C, Supplemental Fig. S5B), ${\dot{{\rm{V}}}}_{{\rm{E}}}$ (−1.9847 mL/min/g, p < 0.0001, Fig. 4D, Supplemental Fig. S5C), and ${\dot{{\rm{V}}}}_{{\rm{E}}}$/${\dot{{\rm{V}}}}_{{{\rm{O}}}_{2}}$ (−40.8103, p < 0.0001, Fig. 4F, Supplemental Fig. S5E). Six females and six males were assayed under the hypercapnic protocol and no significant differences were seen under room air or hypercapnic conditions. Under hypoxic conditions, *Egr2* ^*Cre*^; *P_hM4D* animals showed reduced V_f_ (−38.5233 breaths/min, p = 0.0018, Fig. 5B, Supplemental Fig. S6A), V_T_ (−0.0010 mL/breath/g, p = 0.048, Fig. 5C, Supplemental Fig. S6B), ${\dot{{\rm{V}}}}_{{\rm{E}}}$ (−0.5992 mL/min/g, p < 0.0001, Fig. 5D, Supplemental Fig. S6C), and ${\dot{{\rm{V}}}}_{{\rm{E}}}$/${\dot{{\rm{V}}}}_{{{\rm{O}}}_{2}}$ (−12.5255, p = 0.0054, Fig. 5F, Supplemental Fig. S6E). Six females and six males were assayed under the hypoxic protocol and no significant differences were seen under room air or hypoxic conditions.

#### Acute perturbation of *HoxB1*^*Cre*^ neurons in adult mice results in modified room air breathing and reductions in hypercapnic and hypoxic ventilation with increased periodic and volume instability

As previously described, the *HoxB1* ^*Cre*^ domain captures cells derived from rhombomere 4 and posterior migratory regions, and includes several populations of cells previously implicated in respiratory physiology: a subset of neurons from the RTN, the PreBötzinger Complex, and noradrenergic A5, A2, and A1 nuclei^39^.

Under room air conditions, after CNO administration, *HoxB1* ^*Cre*^; *P_hM4D* animals showed reduced V_f_ (−92.943 breaths/min, p < 0.0001, Fig. 3B, Supplemental Figs S7A and S8A), $\dot{{\rm{V}}}$_E_ (−0.8239 mL/min/g, p = 0.00037, Fig. 3D, Supplemental Figs S7C and S8C), ${\dot{{\rm{V}}}}_{{{\rm{O}}}_{2}}$ (−0.0292 mL/min/g, p < 0.0001, Fig. 3E, Supplemental Figs S7D and S8D), and increased $\dot{{\rm{V}}}$_E_/$\dot{{\rm{V}}}$_O2_ (+10.4035, p = 0.048, Fig. 3F, Supplemental Figs S7E and S8E) as compared to pre-CNO values and sibling controls. Under hypercapnic conditions, after CNO administration, *HoxB1* ^*Cre*^; *P_hM4D* animals showed reduced V_f_ (−149.6239 breaths/min, p < 0.0001, Fig. 4B, Supplemental Fig. S7A), ${\dot{{\rm{V}}}}_{{\rm{E}}}$ (−2.5931 mL/min/g, p < 0.0001, Fig. 4D, Supplemental Fig. S7C), ${\dot{{\rm{V}}}}_{{{\rm{O}}}_{2}}$ (−0.0172 mL/min/g, p < 0.0001, Fig. 4E, Supplemental Fig. S7D), and ${\dot{{\rm{V}}}}_{{\rm{E}}}$/${\dot{{\rm{V}}}}_{{{\rm{O}}}_{2}}$ (−37.6294, p = 0.013, Fig. 4F, Supplemental Fig. S7E), and increased periodic instability (+0.0165 in CV, p = 0.0011, Fig. 4I, Supplemental Fig. S7H) and volume instability (+0.0142 in CV, p < 0.0001, Fig. 4J, Supplemental Fig. S7I). Five females and seven males were assayed under the hypercapnic protocol and no significant differences were seen under room air or hypercapnic conditions. Under hypoxic conditions, *HoxB1* ^*Cre*^ *; P_hM4D* animals had a reduced V_f_ (−101.6985 breaths/min, p < 0.0001, Fig. 5B, Supplemental Fig. S8A), ${\dot{{\rm{V}}}}_{{\rm{E}}}$ (−0.797 mL/min/g, p = 0.00056, Fig. 5D, Supplemental Fig. S8C), ${\dot{{\rm{V}}}}_{{{\rm{O}}}_{2}}$ (−0.0126 mL/min/g, p = 0.0032, Fig. 5E, Supplemental Fig. S8D), and periodic instability (+0.0206 in CV, p < 0.0001, Fig. 5I, Supplemental Fig. S8H) and volume instability (+0.0123 in CV, p = 0.0014, Fig. 5J, Supplemental Fig. S8I). Eight females and five males were assayed under the hypoxic protocol and no significant respiratory differences were seen under room air or hypoxic conditions. Female *HoxB1* ^*Cre*^ *; P_hM4D* animals showed a slightly lower temperature at the end of the hypoxic assay compared to males (33.76 °C vs. 34.94 °C, p = 0.026) but no significant difference was seen 30 minutes later. Overall, *HoxB1* ^*Cre*^ *; P_hM4D* animals also showed a lower temperature as compared to sibling controls at the end of respiratory assays (−2.2797 °C, p < 0.0001) and 30 minutes after the end of the assay (−10.207 °C, p < 0.0001) (Fig. 6, Supplemental Figs S7J and S8J).

#### Acute perturbation of *HoxA4-Cre* neurons in adult mice results in increased room air ventilation and increased minute ventilation and oxygen consumption under hypercapnic conditions

As described previously, the *HoxA4-Cre* domain covers neurons including and posterior to r7, and shows expression in the spinal cord^32^. The nucleus tractis solitaris (NTS) and ventral respiratory column (VRC) are also likely derived from the r7 and r8 domain^40^.

Under room air conditions, after CNO administration, *HoxA4-Cre*; *P_hM4D* animals showed increased V_f_ (+59.685 breaths/min, p < 0.0001, Fig. 3B, Supplemental Figs S9A and S10A), V_T_ (+0.0027 mL/breath, p = 0.00026, Fig. 3C, Supplemental Figs S9B and S10B), ${\dot{{\rm{V}}}}_{{\rm{E}}}$ (+1.1896 mL/min/g, p < 0.0001, Fig. 3D, Supplemental Figs S9C and S10C), ${\dot{{\rm{V}}}}_{{{\rm{O}}}_{2}}$ (+0.0201 mL/min/g, p < 0.0001, Fig. 3E, Supplemental Figs S9D and S10D), and ${\dot{{\rm{V}}}}_{{\rm{E}}}$/${\dot{{\rm{V}}}}_{{{\rm{O}}}_{2}}$ (+7.7544, p = 0.019, Fig. 3F, Supplemental Figs S9E and S10E) as compared to pre-CNO values and sibling controls. Under hypercapnic conditions, after CNO administration, *HoxA4-Cre*; *P_hM4D* animals showed an increased ${\dot{{\rm{V}}}}_{{\rm{E}}}$ (+1.0559 mL/min/g, p = 0.028, Fig. 4D, Supplemental Fig. S9C) mediated by small but statistically insignificant increases in both V_f_ and V_T_, and increased ${\dot{{\rm{V}}}}_{{{\rm{O}}}_{2}}$ (+0.0217 mL/min/g, p < 0.0001, Fig. 4E, Supplemental Fig. S9D) with no change in ${\dot{{\rm{V}}}}_{{\rm{E}}}$/${\dot{{\rm{V}}}}_{{{\rm{O}}}_{2}}$. Seven females and four males were assayed under the hypercapnic protocol and no significant differences were seen under room air or hypercapnic conditions. Under hypoxic conditions, after CNO administration, *HoxA4-Cre*; *P_hM4D* animals showed no change in any parameters as compared to sibling controls (Fig. 5, Supplemental Fig. S10). Nine males and three females were assayed under the hypoxic protocol and no significant respiratory differences were observed under room air conditions. Under hypoxic conditions, females *HoxA4-Cre*; *P_hM4D* animals showed a slightly higher V_T_ than males (0.0116 vs. 0.0076 mL/breath/g, p = 0.0047). Male *HoxA4-Cre*; *P_hM4D* animals showed a significantly reduced temperature 30 minutes at the end of the assay as compared to females (29.44 °C vs. 35.33 °C, p = 0.0041). Overall, *HoxA4-Cre*; *P_hM4D* animals showed a lower temperature 30 minutes after the end of the assay as compared to sibling controls (−4.7261 °C, p < 0.0001, Fig. 6, Supplemental Figs S9J and S10J).

### hM3D DREADD mediated acute stimulation of rhombomeric domains results in rapid death, likely through perturbed respiratory or cardiovascular function

**Figure 7:**
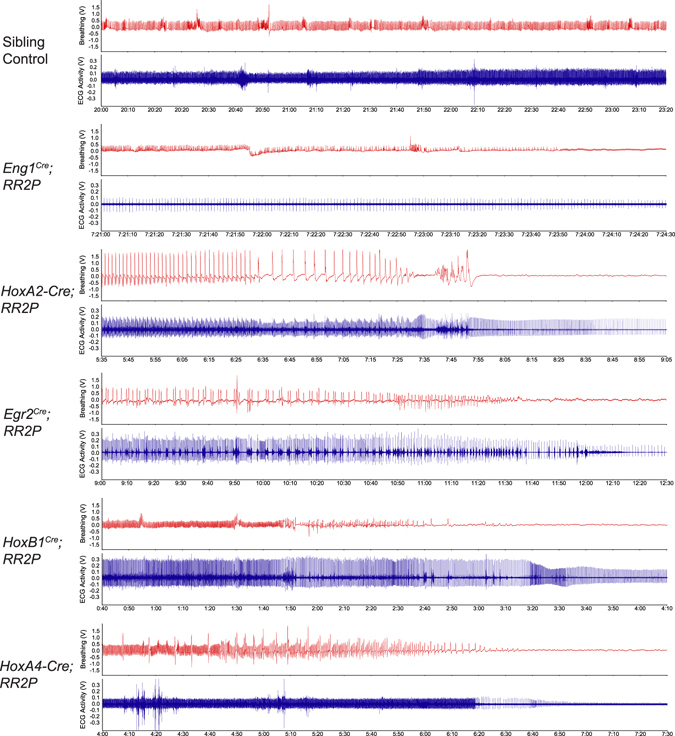
CNO-hM3D perturbation of rhombomeric domains in the adult animal results in death. After CNO injection, *rhombomere-Cre; RR2P* animals die of cardiovascular or respiratory deficiencies. Examination of the respiratory traces and their alignment with electrocardiogram (ECG) heart traces reveals that each of the 5 *rhombomere-Cre; RR2P* models has a different course of death. Time shown under each trace is the time after CNO injection.

After determining that acute inactivation of each rhombomeric domain had dramatic and different respiratory results, we sought to generate a mouse that would acutely activate targeted neuron populations to further examine the role of each rhombomeric domain in respiration. We generated a *Rosa26* knock-in of an intersectional cassette that expresses the modified hM3D activating receptor (*RR2)*. After functional validation, we generated Cre responsive hM3D mice (*RR2P)* by crossing *RR2* mice to the *B6;SJL-Tg(ACTFLPe)9205Dym/J* mouse line (JAX 003800) that ubiquitously expresses FLPe recombinase, thus excising out the FLP-responsive stop cassette. We then bred *Eng1* ^*Cre*^, *HoxA2-Cre*, *Egr2* ^*Cre*^, *HoxB1* ^*Cre*^, and *HoxA4-Cre* animals to the *RR2P* to examine respiratory output upon acute stimulation of the targeted rhombomere domains. Notably, all five animals died after CNO injection, likely due to perturbed respiratory or cardiovascular function. To shed more insight on the mechanism of death, we attached ECG clips to animals to record heart activity simultaneously with respiration (Fig. 7). Examination of the respiratory traces and their alignment with electrocardiogram (ECG) heart traces reveals that each of the 5 *rhombomere-Cre; RR2P* models has a different course of death. *Eng1* ^*Cre*^ *; RR2P* animals had the longest course of death as it took more than eight hours, during which breathing became highly irregular and heart rate increased before slowing down again and final simultaneous cessation of breathing and heart rate. In *HoxA2-Cre; RR2P* animals, breathing slowed down significantly with much larger breaths while heart rate stayed steady with several observations of seizure-like activity, followed by cessation of breathing within 10 minutes of CNO administration and slowing of the heart rate over another 15–20 minutes. In *Egr2* ^*Cre*^ *; RR2P* animals, breathing became irregular and heart rate slowed down shortly after CNO administration, followed by cessation of breathing within 10 minutes and continued slowing of the heart rate for another 10–15 minutes. In *HoxB1* ^*Cre*^ *; RR2P* animals, breathing rate increased while heart rate remained steady, followed by rapid cessation of breathing within 5 minutes and then slowing of the heart rate for 5–10 minutes. Finally, in *HoxA4-Cre*; *RR2P* animals, both breathing and heart rate stayed steady with an increase in the frequency of sighs or gasps (~1 every 20 breaths), until rapid cessation of breathing within 7 minutes and rapid slowing of the heart for another 10–15 minutes.

## Discussion

The specific differences in respiratory function, metabolism and temperature regulation demonstrate that adult domains captured by the expression of early embryonic patterning genes broadly align with anterior-posterior aspects of the adult respiratory network functional organization and define distinct populations that are differentially involved in respiratory, temperature, metabolic, and cardiovascular homeostasis. As a necessity, respiratory networks are patterned during early embryogenesis with very little functional input and feedback (fetal respiratory movements) in preparation for the first breath after birth. Upon birth and in early life these networks are further refined and stabilized as they integrate additional feedback and information from air breathing and feeding. Though this early patterning of homeostatic circuitry is widely presumed to persist into adulthood based on perinatal studies, the contributions of embryonic patterning to mature adult networks is not entirely clear. Our work bypasses embryonic and neonate lethality to directly examine the contributions of neurons defined by the expression of early hindbrain patterning genes in adult respiratory network organization.

Perturbation of the *Eng1* ^*Cre*^ domain under room air conditions resulted in an increase in ${\dot{{\rm{V}}}}_{{{\rm{O}}}_{2}}$ with minimal changes in other respiratory parameters, suggesting that the *Eng1* ^*Cre*^ domain captures populations that are involved in metabolic control. We also observed that breathing became more stable as indicated by reductions in apnea frequency and volume and periodic instability. Considering that the *Eng1* ^*Cre*^ domain captures a majority of the pons which includes nearly the entire locus coeruleus^36^ and likely the parabrachial (PB) and Kolliker-Fuse (KF) nuclei thought to be involved in the inspiratory/expiratory transition^25^, the increase in stability suggests that inhibition of these areas could inadvertently compensate for potential dysfunctional interactions between the pontine and medullary respiratory areas that C57BL/6 J mice may have a genetic predisposition for^41^.

*Eng1* ^*Cre*^ domain perturbation under hypercapnic conditions results in a reduction in V_f_, ${\dot{{\rm{V}}}}_{{\rm{E}}}$, and ${\dot{{\rm{V}}}}_{{\rm{E}}}$/${\dot{{\rm{V}}}}_{{{\rm{O}}}_{2}}$, with no change in ${\dot{{\rm{V}}}}_{{\rm{O2}}}$ as compared to pre-CNO values. This phenotype may arise from perturbation of the locus coeruleus, a noradrenergic nucleus that has been previously implicated in several studies in contributing to the hypercapnic ventilatory response^42^. Under hypoxic conditions, small but insignificant changes in ${\dot{{\rm{V}}}}_{{\rm{E}}}$ and ${\dot{{\rm{V}}}}_{{{\rm{O}}}_{2}}$ resulted in a reduced ${\dot{{\rm{V}}}}_{{\rm{E}}}$/${\dot{{\rm{V}}}}_{{{\rm{O}}}_{2}}$, suggesting that some respiratory phenotypes may be subtle and masked under normal breathing variability among mice and may be revealed only when examining more normalized measurements, such as breathing in ratio to metabolic rate.

The *HoxA2-Cre* domain is likely the smallest cellular population as observed in the embryonic samples and adult brain sections and accordingly, we see no phenotype under room air or hypercapnic conditions. However, under hypoxic conditions, acute inhibition of the *HoxA2-Cre* domain results in enhanced respiration with significant increases in V_f_, V_T_, ${\dot{{\rm{V}}}}_{{\rm{E}}}$, and ${\dot{{\rm{V}}}}_{{\rm{E}}}$/${\dot{{\rm{V}}}}_{{{\rm{O}}}_{2}}$ with no change in ${\dot{{\rm{V}}}}_{{{\rm{O}}}_{2}}$. While a previous study shows that that *HoxA2* ^*-/-*^ neonates have increased V_T_ before they die within 24 hours of birth, few studies have looked at adult expression of r2 derived neurons or respiratory control^26^. These data suggest that *HoxA2-Cre* defined neurons or a smaller subset within could potentially be a therapeutic target for disorders with abnormal responses to hypoxia such as Parkinson’s disease or Rett Syndrome.

In the *Egr2* ^*Cre*^ domain, we build upon existing data that showed that acute perturbation using the same CNO-hM4D system resulted in a reduction in V_f_, V_T_, and $\dot{{\rm{V}}}\,$_E_ under hypercapnic conditions^27^. We see the same hypercapnic deficits and newly report ${\dot{{\rm{V}}}}_{{\rm{O2}}}$ and ${\dot{{\rm{V}}}}_{{\rm{E}}}$/${\dot{{\rm{V}}}}_{{{\rm{O}}}_{2}}$, which is also blunted after CNO administration. Interestingly, we observe a room air ventilation deficit not reported in the previous study (in V_T_, ${\dot{{\rm{V}}}}_{{\rm{E}}}$, and ${\dot{{\rm{V}}}}_{{\rm{E}}}$/${\dot{{\rm{V}}}}_{{{\rm{O}}}_{2}}$) and show that acute perturbation of the rhombomeres 3 and 5 domain also results in a reduction in the hypoxic ventilatory response (in V_f_, V_T_, ${\dot{{\rm{V}}}}_{{\rm{E}}}$, and ${\dot{{\rm{V}}}}_{{\rm{E}}}$/${\dot{{\rm{V}}}}_{{{\rm{O}}}_{2}}$). A subset of serotonergic neurons derived from the *Egr2* ^*Cre*^ domain has been suggested to contribute to the full hypercapnic ventilatory response, though importantly, only $\dot{{\rm{V}}}\,$_E_ was reported and not ${\dot{{\rm{V}}}}_{{\rm{E}}}$/${\dot{{\rm{V}}}}_{{{\rm{O}}}_{2}}$ ^26^. Also, a majority of the retrotrapezoid nucleus (RTN, also well-characterized in its involvement in chemosensation) is likely captured by the driver, but additional yet to be identified neuron populations may contribute to baseline respiration and the hypoxic ventilatory response in the *Egr2* ^*Cre*^ domain.

The *HoxB1* ^*Cre*^ domain is initiated in rhombomere 4 and early developmental studies have shown that a subset of r4 postmitotic neurons migrates posteriorly and that additional posterior mosaic expression is found^31, 43, 44^. Previous studies indicate that the driver captures a subset of many nuclei implicated in respiratory control, including the RTN, A1, A2, and A5 noradrenergic nuclei, and PreBötzinger Complex involved in inspiratory rhythm generation (although with mosaic expression in each nucleus). A lack of full respiratory arrest may be attributable to this mosaic expression failing to silence sufficient numbers of PreBötzinger Complex neurons^45^. A previous study demonstrated that loss of MecP2 in *HoxB1* ^*Cre*^ neurons results in a moderately increased V_T_ under baseline conditions and increased V_f_ under hypoxic conditions^39^. Here we see that perturbation under room air conditions results in major reductions in V_f_, $\dot{{\rm{V}}}\,$_E_, and a proportionally larger decrease in $\dot{{\rm{V}}}\,$_O2_ that results in an increased $\dot{{\rm{V}}}\,$_E_/$\dot{{\rm{V}}}\,$_O2_, suggesting a major metabolic role for this domain. Under hypercapnic conditions, we see a near loss of the hypercapnic ventilatory response with major decreases in V_f_, ${\dot{{\rm{V}}}}_{{\rm{E}}}$, ${\dot{{\rm{V}}}}_{{{\rm{O}}}_{2}}$, and here, since the decrease in $\dot{{\rm{V}}}\,$_O2_ is proportionally smaller than the decrease in ${\dot{{\rm{V}}}}_{{\rm{E}}}$, a reduced ${\dot{{\rm{V}}}}_{{\rm{E}}}$/${\dot{{\rm{V}}}}_{{{\rm{O}}}_{2}}$. Under hypoxic conditions, we see a significant decrease in V_f_, ${\dot{{\rm{V}}}}_{{\rm{E}}}$, ${\dot{{\rm{V}}}}_{{{\rm{O}}}_{2}}$, with no change in ${\dot{{\rm{V}}}}_{{\rm{E}}}$/${\dot{{\rm{V}}}}_{{\rm{O2}}}$, demonstrating how some ventilatory changes may be driven by changes in metabolism.

After *HoxB1* ^*Cre*^ perturbation, under both hypercapnia and hypoxia, we see significant increases in periodic and volume instability. As respiratory pattern is often altered in respiratory disorders including Rett Syndrome, central sleep apneas, and Cheyne-Stokes respiration, this domain may capture cellular populations involved in pattern control, such as the PreBötzinger Complex^46^, and postinspiratory complex (PiCo)^47^. Closer waveform analysis suggests the PiCo complex may be disrupted as waveforms lack the distinctive secondary expiration slope seen in wildtype traces.

Finally, data from the *HoxA4-Cre* domain further highlight the importance of concurrent measurement of oxygen consumption (${\dot{{\rm{V}}}}_{{\rm{O2}}}$) during respiratory studies. While perturbation of the *HoxA4-Cre* population increases all respiratory parameters under room air conditions, perturbation under hypercapnic conditions significantly increases ${\dot{{\rm{V}}}}_{{\rm{E}}}$ while ${\dot{{\rm{V}}}}_{{\rm{O2}}}$ increases proportionally, resulting in an unchanged ${\dot{{\rm{V}}}}_{{\rm{E}}}$/${\dot{{\rm{V}}}}_{{{\rm{O}}}_{2}}$ ratio, which suggests that the affected cells may also be involved in inhibition of arousal, sympathetic activity, and metabolism and that the respiratory increases under hypercapnia may be secondary to the increase in metabolism.

*Eng1* ^*Cre*^ *; P_hM4D, HoxA2-Cre; P_hM4D, HoxB1* ^*Cre*^ *; P_hM4D*, and *HoxA4-Cre*; *P_hM4D* animals all showed significant temperature deficits 30 minutes after removal from the chamber (~1.5 hours after CNO injection), with *HoxB1* ^*Cre*^ *; P_hM4D* animals showing the most dramatic drop that was also significant immediately at the end of the respiratory assays (~1 hour after CNO injection), in spite of the chamber temperature being held at 30–31°C (within the thermo-neutral zone). Thermogenesis is an essential component of physiological homeostasis in maintaining temperature between 35 and 39°C in mammals. Anatomical and functional studies have indicated that the rostral ventromedial medullary neurons play a role of central thermogenesis^48^, and other brainstem areas express temperature-sensitive receptors or receive projections from the hypothalamus, thought to be a control center for central thermoregulation^49^. Further refinement of these neural subtypes may shed insight on therapeutics for metabolic disorders such as obesity.

Assessment of temperature, ${\dot{{\rm{V}}}}_{{\rm{O2}}}$ and ${\dot{{\rm{V}}}}_{{\rm{E}}}$/${\dot{{\rm{V}}}}_{{{\rm{O}}}_{2}}$ reveals that metabolic changes underlie changes in baseline and chemosensory respiratory phenotypes. Proportionate changes suggest that a simple increase in metabolic demand mediates an increase in ${\dot{{\rm{V}}}}_{{\rm{E}}}$ while disproportionate changes leading to perturbed baseline ${\dot{{\rm{V}}}}_{{\rm{E}}}$/${\dot{{\rm{V}}}}_{{{\rm{O}}}_{2}}$ may then result in respiratory alkalosis or acidosis. For example, we see that perturbation of the *HoxB1* ^*Cre*^ *; P_hM4D and HoxA4-Cre*; *P_hM4D* populations results in an increased ventilation to oxygen consumption ratio, suggesting that the animals may be hypocapnic as they enter the high CO_2_ respiratory challenge. Thus, the ventilatory deficit in the *HoxB1* ^*Cre*^ *; P_hM4D* animals may actually be more pronounced than we observe and a deficit in the *HoxA4-Cre*; *P_hM4D* animal may exist but could be masked due to a portion of the hypercapnic challenge acting to restore blood P_CO2_ to baseline resulting in an effective smaller hypercapnic challenge. In contrast, the *Egr2* ^*Cre*^; *P_hM4D* animals have a lower ${\dot{{\rm{V}}}}_{{\rm{E}}}$/${\dot{{\rm{V}}}}_{{{\rm{O}}}_{2}}$ under baseline conditions and may be hypercapnic as they enter the high CO_2_ challenge. As previous studies suggest that the ${\dot{{\rm{V}}}}_{{\rm{E}}}$ response to increasing levels of CO_2_ does not necessarily follow a linear curve^50, 51^, the subsequent ventilatory deficit may not accurately reflect the magnitude of the respiratory phenotype. Our findings show the importance of measuring $\dot{{\rm{V}}}$_O2_ in acute circuit perturbation studies to separate primary and secondary respiratory phenotypes.

In Sudden Unexpected Death in Epilepsy (SUDEP), patients with epilepsy die unexpectedly after seizures, commonly while sleeping. One hypothesis is that SUDEP results from perturbed autonomic function, including the disruption of key brainstem circuits, from spreading depolarization that may start in the brainstem or in other areas of the brain and spread to the brainstem^11^. Considering that CNO-hM3D mediated activation of rhombomere domains resulted in differential paths to death for all five experimental crosses, our experimental hM3D animals may serve as possible models of SUDEP for further mapping studies to shed insight upon which neural populations may be vulnerable to brainstem spreading depolarization as well as the role of repeated sub lethal events using smaller doses of CNO.

As we interpret the results, several experimental considerations must be discussed. First, the rhombomeric domains mapped out here are defined by Cre drivers that are either targeted to the locus of a rhombomere specific gene or utilize enhancer and promoter elements from such a gene. As either approach can result in perturbed or incomplete transcriptional, translational, and post-translational control, Cre activity may or may not fully recapitulate the temporal - spatial expression of the endogenous gene and presumed rhombomeric domains. We see in Fig. 2 that both during embryogenesis and in the adult, our repertoire of widely used Cre drivers show activity outside of the brainstem in other regions of the CNS including the forebrain and in the spinal cord. As these drivers are expressed during neurogenesis, we must presume that we have also captured later derived glial populations. Additionally, it is possible and likely that these drivers capture migrating neural crest and its derivatives including components of the sympathetic and parasympathetic neural systems and may have expression in the enteric system and also non-neural tissues. This must also be taken into account as markers used in intersectional mapping may show expression outside of the CNS in tissues that affect homeostasis, pH, and metabolism^52^. However, many of our phenotypes do align to prior anatomically based functional studies of populations that reside within specific rhombomeric domains and the overlapping posterior expression domains of *HoxB1* ^*Cre*^ and *HoxA4-Cre* that show notably distinct phenotypes suggest that the observed phenotypes are largely the result of perturbations to brainstem circuitry. Nonetheless, if additional central and peripheral populations captured by these drivers do play a role in these phenotypes, it raises the possibility that these genes and their corresponding drivers capture broad developmental modules that coordinately pattern brainstem and peripheral populations into coordinated homeostatic networks. Similar patterning roles have been suggested for *Phox2b* ^53^.

Second, the ability of targeted cells to respond to either hM3D and hM4D activation and the cell specific consequence of that activation cannot be readily assessed in this study due to the size, diversity, and complexity of the individual developmental rhombomeric populations in the adult. Graded DREADD expression reflective of Cre variability is not expected as the DREADDs are expressed from the Rosa/CAG promoter in a binary fashion once a Cre threshold for recombination is reached. Specific cell types, though, may not efficiently express the Rosa/CAG construct. Significant variability in cellular responses to DREADD activation have been seen in other studies and likely relates to expression differences of endogenous downstream Gi and Gq signaling pathway components^33^. While there is concern that the level of CNO used in these studies may be high, these and previous studies have shown that sibling control mice that do not express DREADD receptors have equivalent respiratory parameters pre- and post–CNO exposure; thus the experiments are well controlled within (room air and hypercapnic conditions) and between groups (room air, hypercapnic, and hypoxic conditions). In our studies all mice were naïve to CNO and used only once, ruling out the possibility that repeated CNO exposures or DREADD activation might affect the observed phenotype as was seen for body temperature in serotonergic DREADD perturbations^33^.

In conclusion, the anatomical and functional fate maps generated in this study support the notion that embryonic patterning remains a defining feature in adult homeostatic circuit organization, which is likely further shaped by additional external cues occurring as the animal transitions from an amniotic environment and matures into adulthood. The DREADD mouse models can be further used to test this idea by examining if perturbation of these embryonic domains at various postnatal stages leads to disturbances in respiratory maturation. Finally, these data provide a framework and genetic marker candidates for more refined intersectional fate mapping for circuits important in SIDS and SUDEP.

## Materials and Methods

### Ethical Approval

Studies were approved by the Baylor College of Medicine Institutional Animal Care and Use Committee under protocol AN-6171 and all experiments were performed in accordance with relevant guidelines and regulations.

### Generation of the RR2 intersectional mouse

To generate the *RR2* mouse line that expresses the hM3D activating DREADD in a FLP/Cre responsive manner, we created a targeting vector with a 1 kb 5′ homology arm and 5 kb 3′ homology arm to the *Rosa26* locus that consisted of: 1) a ubiquitous *CAG* promoter; 2) an *FRT*-flanked stop cassette; 3) a *LoxP*-flanked mCherry and stop cassette; and 4) the hM3D cDNA^34^.

Embryonic stem (ES) cells (Ab2.2) were electroporated with the targeting vector. Neomycin selected clones were screened for homologous recombination using PCR genotyping for 5′ and 3′ targeting from outside the homology arm. We used pairs 5′GGGCGTACTTGGATATGAT (Rosa26-F) and 5′CGCCTAAAGAAGAGGCTGTG (CAG-R), producing a 1472 bp band, and 5′AATCAACCTCTGGATTACAAAATTT (WPRE-F) and 5′TCTCCCCTCAGAGAAATGGA (Rosa26-R), producing a 5254 bp band. Selected clones were microinjected into C57B1/J6 blastocysts and chimeric males were bred to wildtype C57B1/J6 females to achieve germline transmission. To generate mice that expressed the hM3D activating DREADD in a Cre responsive manner (*RR2P)*, mice were crossed with B6;SJL-Tg(ACTFLPe)9205Dym/J mice (JAX 003800) to permanently delete the *FRT* flanked stop cassette.

### Breeding, Genetic Background, and Maintenance of Mice

We maintained colonies of all heterozygous mouse strains by backcrossing to C57BL/6 J mice, and homozygous strains by sibling crosses. For histology experiments, *Eng1* ^*Cre*^ ^28^,﻿ *HoxA2-Cre* ^29^, *Egr2* ^*Cre*^ ^30^, *HoxB1* ^*Cre*^ ^31^, and *HoxA4-Cre* ^32^ mice were mated with homozygous Ai9^54^ mice (JAX 007909). For plethysmography experiments, the same *Cre* drivers were mated with homozygous *RC::P_hM4D* ^33^ or *RR2P* mice to derive animals in which all mice carried the *RC::P_hM4D* or *RR2P* alleles. Sibling animals that did not inherit the *Cre* allele were used as control animals to the *Cre* positive offspring. *Rosa26* specific primers for the *Ai9*, *RC::P_hM4D*, and *RR2P* mice were 5′-GCACTTGCTCTCCCAAAGTC, 5′-GGGCGTACTTGGCATATGAT, and 5′-CTTTAAGCCTGCCCAGAAGA, and yield a 495 bp band (targeted) and 251 bp band (wt). Cre-specific primers for all the rhombomere Cre drivers were 5′-ATCGCCATCTTCCAGCAGGCGCACCATTGCCC and 5′-GCATTTCTGGGGATTGCTTA and yielded a 550 bp band if positive.

### Plethysmography

Plethysmography on conscious, unrestrained mice was carried out as described on 6–12 week old adult animals^33^. Briefly, mice were taken from their home cage, weighed, and rectal temperature was taken. Animals were then placed into an airtight, temperature controlled (32 °C) plethysmography chamber and allowed to acclimate for at least 20 minutes in room air (21% O_2_/79% N_2_) conditions. For the hM3D experiments, ECG clips were attached to animals for heart rate read-out. All animals were naïve to clozapine-N-oxide (CNO) and used only once.

#### Hypercapnic Assay

For the hypercapnic assay, after acclimation and measurement under room air, the chamber gas was switched to a hypercapnic mixture of 5% CO_2_/21% O_2_/74% N_2_ for 20 minutes. Chamber gas was then switched back to room air for 20 minutes. The mice were briefly removed for rectal temperature measurement and intra-peritoneal injection of CNO (National Institute of Mental Health Chemical Synthesis and Drug Supply Program) dissolved in saline (1 mg/mL) for an effective concentration of 10 mg/kg. The animal was returned to the chamber for another 20 minutes of room air, 20 minutes of hypercapnia, and 20 minutes of room air. The animal was then removed from the chamber and rectal temperature was taken immediately afterwards and again 30 minutes after the termination of the experiment. The animal was placed in its own cage during these 30 minutes at the ambient room temperature (~23 °C).

#### Hypoxic Assay

For the hypoxic assay, no pre-CNO measurement of hypoxic ventilation was taken due to potential confounds of hypoxic plasticity on the post-hypoxic response. Instead, after acclimation and measurement under room air, mice were removed from the chamber and given 10 mg/kg of CNO. The animal was then returned to the chamber where they were exposed to 20 minutes of room air and 20 minutes of hypoxia (10% O_2_/90% N_2_). The animal was then removed from the chamber and rectal temperature was taken immediately afterwards and again 30 minutes after the termination of the experiment.

### Data Collection and Analysis

Plethysmography pressure changes were measured using a Validyne DP45 differential pressure transducer and reference chamber and CD15 carrier demodulator and recorded with LabChartPro in real time. Waveforms were analyzed offline using LabChartPro and custom written MATLAB code to determine respiratory rate (V_f_), tidal volume (V_T_)^33^, minute ventilation (${\dot{{\rm{V}}}}_{{\rm{E}}}$), and pattern analysis. Respiratory waveforms were selected during periods when the animal was at rest and readings were free from movement artifacts. A minimum of 1 minute of cumulative data compiled from traces at least 10 seconds long from the last 10 minutes of a given experimental condition were analyzed^27, 33^. O_2_ consumption was determined by comparing the gas composition between calibration in an empty chamber and live breathing using an AEI oxygen sensor and analyzer. Using acute injections of a 5%/95% carboxygen gas mixture, a time constant of ~1.5 seconds between the plethysmograph chamber and the inline sensors was calibrated. Chamber temperature was constantly monitored using a ThermoWorks MicroThermo 2 and probe and with LabChartPro in real time.

Poincaré measurements and sigh and apnea frequency were determined using 1 minute of movement-free traces from each breathing condition. Sighs were defined as a breath with an amplitude at least twice as large as the average breath. Apneas were defined as an interbreath interval (IBI) at least twice as large as the average IBI. The coefficient of variation (CV) of the IBI and amplitude was also calculated from the same 1-minute trace compilation of each breathing condition (standard error IBI or amplitude/mean IBI or amplitude).

Room air measurements were combined from both the hypercapnic and hypoxic assay for data shown in Fig. 3. Sibling control data from all five experimental crosses were combined in Figs. 3 and 4 for graph simplification, but statistical significance indicated on the graphs and numerical changes in the Results section was determined using only sibling control data from a given experimental cross. Temperature data was also combined from both the hypercapnic and hypoxic assays in Fig. 6. Individual data from each experimental cross and assay can be seen in Supplemental Figs S1–10.

### Statistics

Results (V_f_, V_T_, ${\dot{{\rm{V}}}}_{{\rm{E}}}$, ${\dot{{\rm{V}}}}_{{{\rm{O}}}_{2}}$, ${\dot{{\rm{V}}}}_{{\rm{E}}}$/${\dot{{\rm{V}}}}_{{{\rm{O}}}_{2}}$, number of apneas and sighs, and CVs of IBI and amplitude) for room air and hypercapnic data were compared between all cohorts using a linear mixed-effects regression model with animal type (experimental or control) and CNO administration (pre or post injection) as fixed effects and animal ID as a random effect. The room air measurements in the hypercapnic and hypoxic assays were pooled together for statistical analysis in Fig. 3 but individual data and statistical significance for each assay can be seen in Supplemental Figs S1–10. Some cohort-to-cohort variability was observed in pre-CNO values in individual assays between experimental and sibling controls groups, but the differences did not remain after data for both assays were pooled. Hypoxic and temperature data were compared using a linear mixed-effects regression model with animal type (experimental or control) as a fixed effect. Sex differences for room air and hypercapnic conditions were compared using a linear mixed-effects regression model with animal type (experimental or control), CNO administration (pre or post) and sex (male or female) as fixed effects and animal ID as a random effect. Sex differences for the hypoxic condition were compared using a linear mixed-effects regression model with animal type and sex as fixed effects. The p-values reported correspond to statistical significance of the conditional interaction between fixed effects. A p-value of <0.05 was used to indicate statistical significance and standard error of the mean is shown on all charts.

### Histology

For embryonic tissue collection, E10.5 embryos were cleared using a modified CLARITY protocol^55^. The embryos were fixed in a 4% acrylamide, 0.05% bis, 0.25% w/v VA-044 initiator, 4% paraformaldehyde (PFA) in 0.1 M phosphate buffered saline (PBS) solution and incubated for 2 hours at 4°C. The embryos were then switched to the acrylamide-bis solution without PFA and incubated overnight at 4°C. After fixation, the embryos were hydrogelled as described^55^ and then passively cleared in an 8% sodium dodecyl sulfate (SDS) in PBS solution at 37 °C for 2–3 days. After clearing, the embryos were then washed with PBS and switched to ScaleA2 clearing agent^56^ and incubated for 2 hours-overnight at room temperature followed by imaging on a Zeiss epifluorescent stereoscope.

For adult tissue collection, 4–8 week old adult mice were transcardially perfused with PBS followed by 4% PFA in PBS. Brains were dissected out and drop fixed in 4% PFA for 2 hours at 4°C followed by a PBS rinse and equilibration in 20% sucrose in 0.1 M PBS. Brains were then cryosectioned into 30 μm sections and mounted on slides. Images were collected on a Zeiss epifluorescent stereoscope. Cell nuclei were visualized with DAPI.

### Data availability

All data generated or analyzed during this study are included in this published article (and its Supplemental Information files). The *RR2* line is publicly available from the MMRRC (Mutant Mouse Regional Resource Centers) as strain MMRRC:043515.

## Electronic supplementary material

Supplementary Information
