# Supplementary material for: Embryonic hindbrain patterning genes delineate distinct cardio-respiratory and metabolic homeostatic populations in the adult
Source: Sci Rep. 2017 Aug 22;7:9117. doi: 10.1038/s41598-017-08810-4 (PMC5567350; doi:10.1038/s41598-017-08810-4)
Supplement: Supplementary file 1 — Supplementary Information [file 41598_2017_8810_MOESM1_ESM.pdf]

TITLE: Embryonic hindbrain patterning genes delineate distinct cardio-respiratory and metabolic homeostatic populations in the adult.

ARTICLE TYPE: Research Article

AUTHOR NAMES AND AFFILIATIONS:

Jenny J. Sun[1], Russell S. Ray[1,*]

[1]Baylor College of Medicine, Department of Neuroscience
1 Baylor Plaza, Houston Texas, 77030
+1 713 798 2624

[*]Corresponding author: russell.ray@bcm.edu

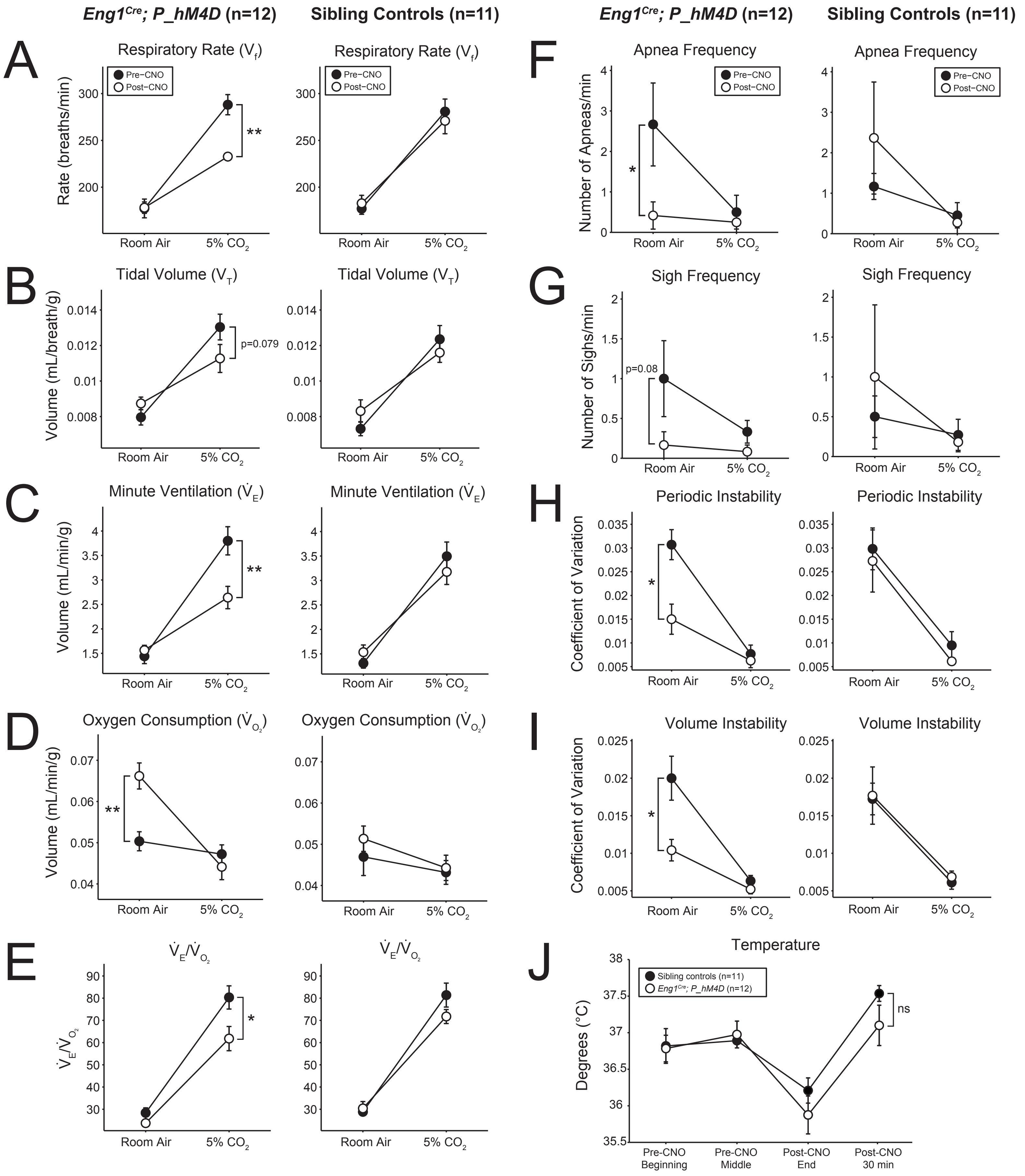

Eng1Cre; P_hM4D (n=12)
Sibling Controls (n=11)
Eng1Cre; P_hM4D (n=12)
Sibling Controls (n=11)
A
Respiratory Rate (Vf)
Rate (breaths/min)
Pre−CNO
Post−CNO
**
Room Air
5% CO2
B
Tidal Volume (VT)
Volume (mL/breath/g)
p=0.079
C
Minute Ventilation (V̇E)
Volume (mL/min/g)
**
D
Oxygen Consumption (V̇O2)
Volume (mL/min/g)
**
E
V̇E/V̇O2
*
F
Apnea Frequency
Number of Apneas/min
*
G
Sigh Frequency
Number of Sighs/min
p=0.08
H
Periodic Instability
Coefficient of Variation
*
I
Volume Instability
Coefficient of Variation
*
J
Temperature
Degrees (°C)
Sibling controls (n=11)
Eng1Cre; P_hM4D (n=12)
ns
Pre-CNO Beginning
Pre-CNO Middle
Post-CNO End
Post-CNO 30 min

Caption: Supplemental Figure S1. Acute perturbation of *Engl^{Cre}* neurons in adult mice in a hypercapnic protocol resulted in increased oxygen consumption and reductions in apnea frequency, periodic instability and volume instability under room air conditions, and reduced respiratory rate, minute ventilation, and $\dot{V}_E/\dot{V}_{O2}$ under hypercapnic conditions. Baseline respiratory parameters were measured pre-CNO under room air and hypercapnic (5% CO_2) conditions, followed by CNO injection (10 mg/kg) and another exposure to room air and hypercapnia. After CNO administration, under room air conditions *Engl^{Cre}; P_hM4D* animals showed increases in oxygen consumption **(D)** and reductions in apnea frequency **(F)**, periodic instability **(H)** and volume instability **(I)** with no significant changes in respiratory rate **(A)**, tidal volume **(B)**, minute ventilation **(C)**, $\dot{V}_E/\dot{V}_{O2}$ **(E)**, or sigh frequency **(G)** as compared to pre-CNO measurements and sibling controls. Under hypercapnic conditions, *Engl^{Cre}; P_hM4D* animals showed a reduced rate **(A)**, minute ventilation **(C)**, and $\dot{V}_E/\dot{V}_{O2}$ **(E)** with no significant changes in other respiratory parameters. No significant changes were seen in temperature **(J)**. Statistical significance was determined using a linear mixed-effects regression model with animal type (experimental or control) and CNO treatment (pre or post) as fixed effects and animal ID as a random effect. *$p<0.05$, **$p<0.01$, ***$p<0.001$.

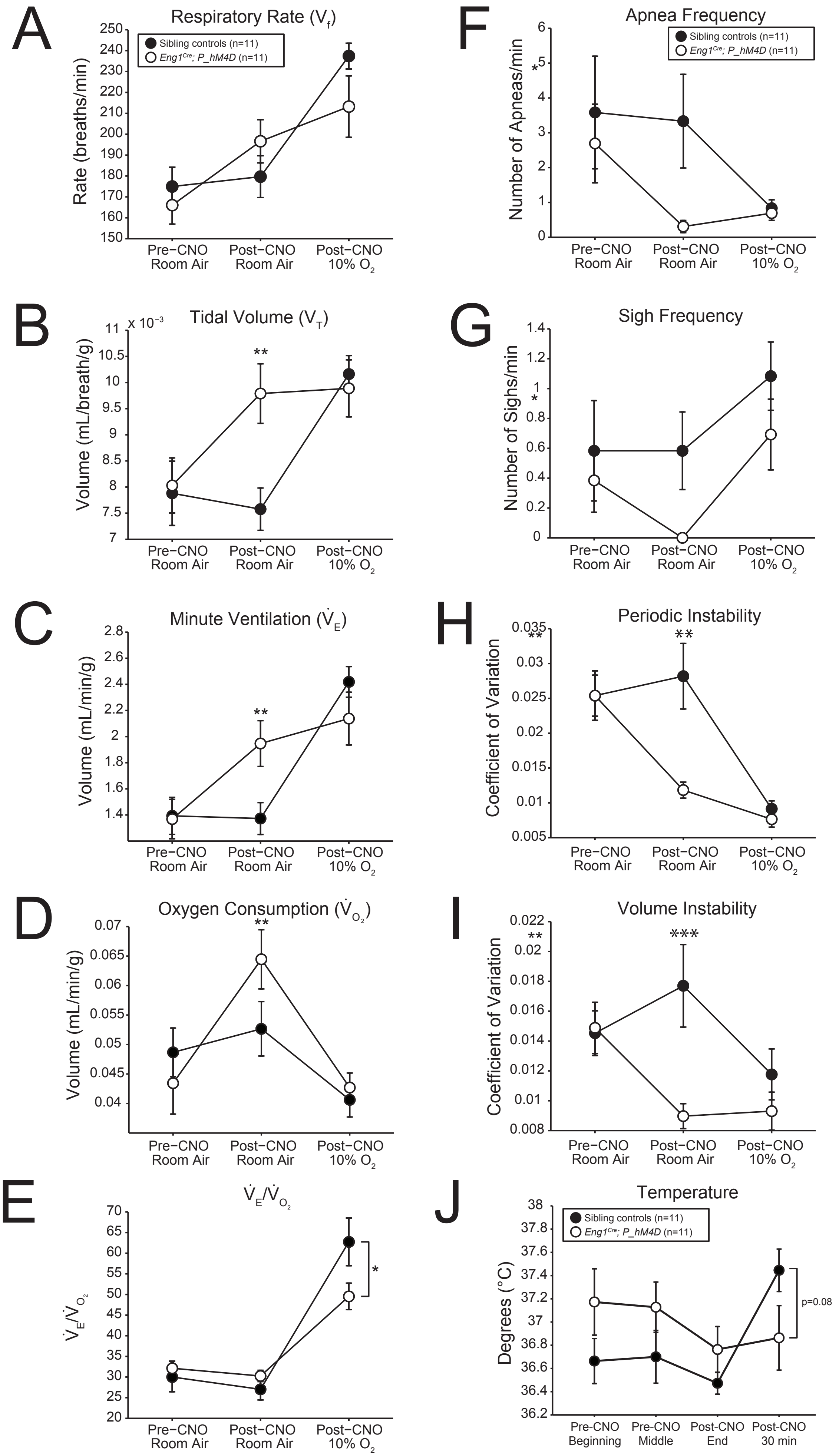
A
Respiratory Rate (V_f)
Rate (breaths/min)
Sibling controls (n=11)
Eng1^{Cre}; P_hM4D (n=11)
Pre−CNO Room Air
Post−CNO Room Air
Post−CNO 10% O_2
B
Tidal Volume (V_T)
x 10^{-3}
Volume (mL/breath/g)
**
C
Minute Ventilation ($\dot{V}_E$)
Volume (mL/min/g)
**
D
Oxygen Consumption ($\dot{V}_{O_2}$)
Volume (mL/min/g)
**
E
$\dot{V}_E/\dot{V}_{O_2}$
*
F
Apnea Frequency
Number of Apneas/min
*
G
Sigh Frequency
Number of Sighs/min
*
H
Periodic Instability
Coefficient of Variation
**
I
Volume Instability
Coefficient of Variation
***
J
Temperature
Degrees (°C)
p=0.08
Pre-CNO Beginning
Pre-CNO Middle
Post-CNO End
Post-CNO 30 min

Caption: Supplemental Figure S2. Acute perturbation of *Engl^{Cre}* neurons in adult mice in a hypoxic protocol resulted in increased tidal volume, minute ventilation and oxygen consumption and reductions in periodic instability and volume instability under room air conditions, and reduced $\dot{V}_E/\dot{V}_{O2}$ under hypoxic conditions. Baseline room air respiratory parameters were measured pre-CNO, followed by CNO injection (10 mg/kg) and another exposure to room air and a single exposure to hypoxia (10% O_2). No pre-CNO 10% O_2 measurement was made to avoid potential confounds of hypoxic plasticity on the post-hypoxic response. After CNO administration, under room air conditions *Engl^{Cre}; P_hM4D* animals showed increases in tidal volume **(B)**, minute ventilation **(C)**, and oxygen consumption **(D)** and reductions in periodic instability **(H)** and volume instability **(I)** with no significant changes in respiratory rate **(A)**, $\dot{V}_E/\dot{V}_{O2}$ **(E)**, or apnea **(F)** or sigh frequency **(G)** as compared to pre-CNO measurements and sibling controls. Under hypoxic conditions, *Engl^{Cre}; P_hM4D* animals showed a reduced $\dot{V}_E/\dot{V}_{O2}$ **(E)** with no significant changes in any other respiratory parameters. No significant changes were seen in temperature **(J)**. Statistical significance was determined using a linear mixed-effects regression model with animal type (experimental or control) and CNO treatment (pre or post) as fixed effects and animal ID as a random effect. *p<0.05, **p<0.01, ***p<0.001.

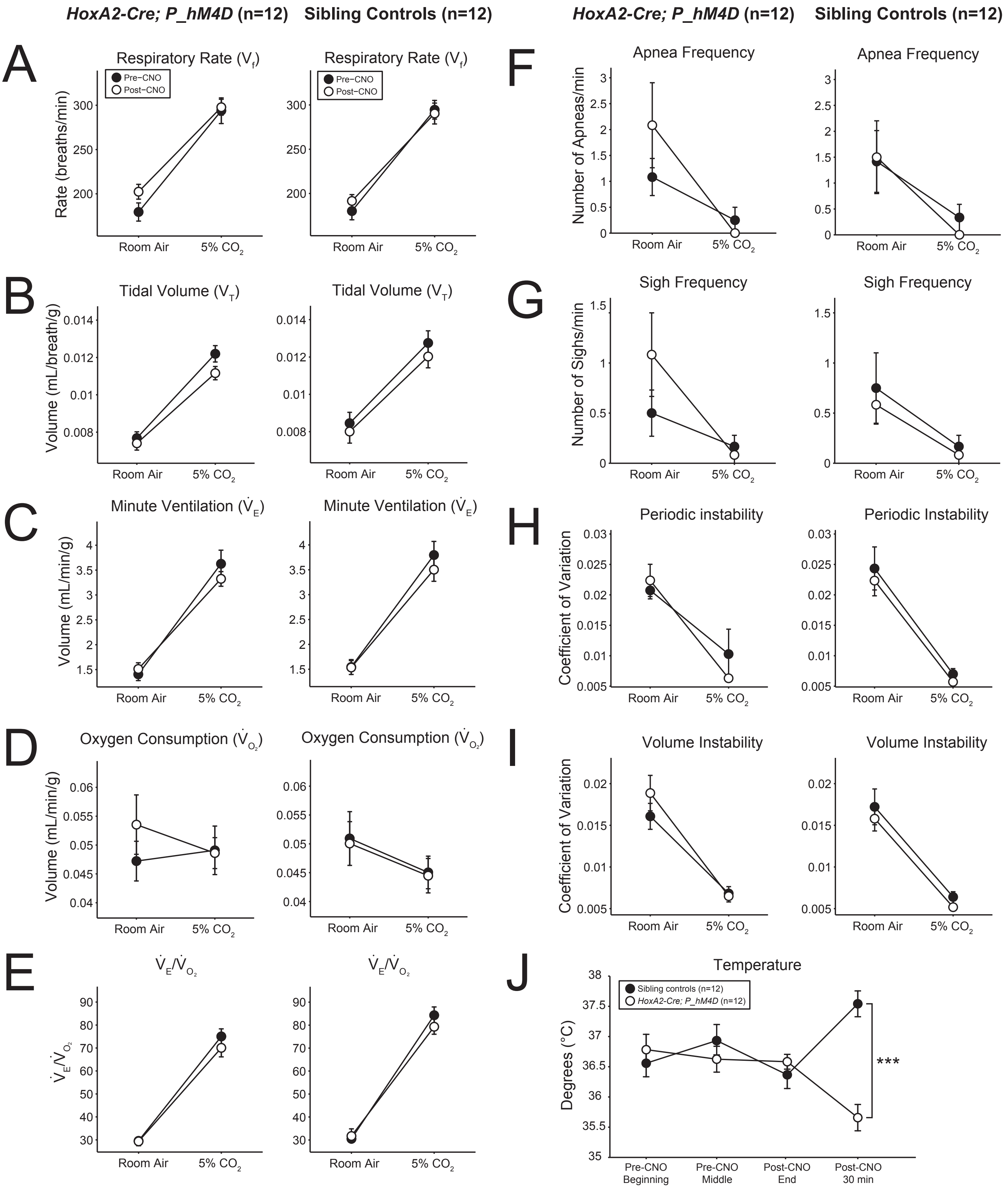

HoxA2-Cre; P_hM4D (n=12)
Sibling Controls (n=12)
HoxA2-Cre; P_hM4D (n=12)
Sibling Controls (n=12)
A
Respiratory Rate (V_f)
Pre−CNO
Post−CNO
Rate (breaths/min)
Room Air
5% CO_2
B
Tidal Volume (V_T)
Volume (mL/breath/g)
C
Minute Ventilation ($\dot{V}_E$)
Volume (mL/min/g)
D
Oxygen Consumption ($\dot{V}_{O_2}$)
Volume (mL/min/g)
E
$\dot{V}_E/\dot{V}_{O_2}$
F
Apnea Frequency
Number of Apneas/min
G
Sigh Frequency
Number of Sighs/min
H
Periodic instability
Periodic Instability
Coefficient of Variation
I
Volume Instability
Coefficient of Variation
J
Temperature
Sibling controls (n=12)
HoxA2-Cre; P_hM4D (n=12)
Degrees (°C)
***
Pre-CNO Beginning
Pre-CNO Middle
Post-CNO End
Post-CNO 30 min

Caption: Supplemental Figure S3. Acute perturbation of *HoxA2-Cre* neurons in adult mice in a hypercapnic protocol resulted in no respiratory changes and a reduced body temperature 30 minutes after the end of the assay. Baseline respiratory parameters were measured pre-CNO under room air and hypercapnic (5% CO_2) conditions, followed by CNO injection (10 mg/kg) and another exposure to room air and hypercapnia. After CNO administration, under both room air and hypercapnic conditions *HoxA2-Cre; P_hM4D* animals showed no significant change in respiratory rate **(A)**, tidal volume **(B)**, minute ventilation **(C)**, oxygen consumption **(D)**, $\dot{V}_E/\dot{V}_{O2}$ **(E)**, apnea **(F)** and sigh frequency **(G)**, or periodic **(H)** and volume instability **(I)** as compared to pre-CNO values and sibling controls. *HoxA2-Cre; P_hM4D* animals did show a significant reduction in temperature 30 minutes after the end of the respiratory assay as compared to sibling controls **(J).** Statistical significance was determined using a linear mixed-effects regression model with animal type (experimental or control) and CNO treatment (pre or post) as fixed effects and animal ID as a random effect. *$p<0.05$, **$p<0.01$, ***$p<0.001$.

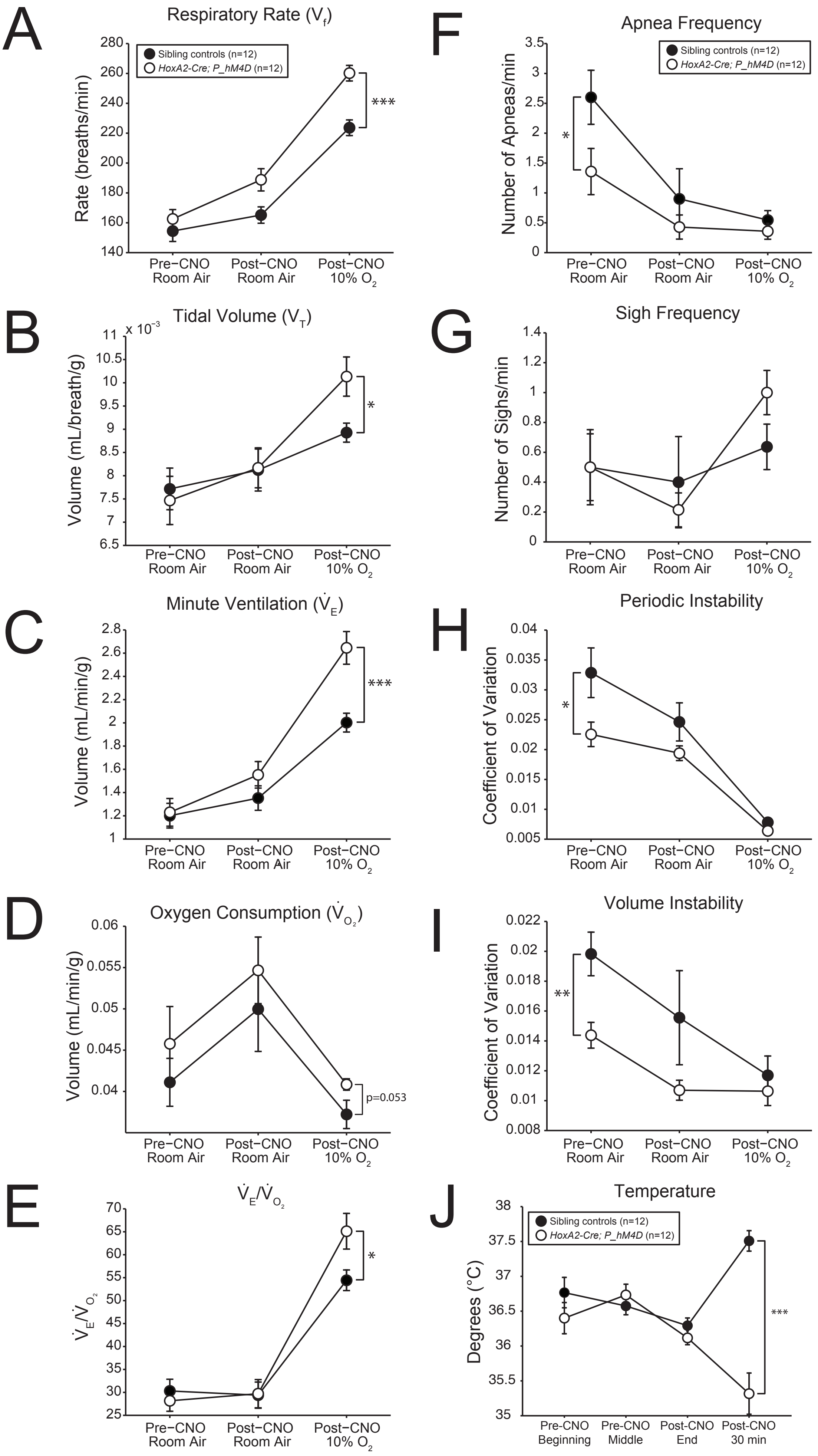
A
Respiratory Rate (Vf)
Sibling controls (n=12)
HoxA2-Cre; P_hM4D (n=12)
Rate (breaths/min)
***
Pre−CNO Room Air
Post−CNO Room Air
Post−CNO 10% O2
B
Tidal Volume (VT)
x 10−3
Volume (mL/breath/g)
*
C
Minute Ventilation (V̇E)
Volume (mL/min/g)
***
D
Oxygen Consumption (V̇O2)
Volume (mL/min/g)
p=0.053
E
V̇E/V̇O2
*
F
Apnea Frequency
Number of Apneas/min
*
G
Sigh Frequency
Number of Sighs/min
H
Periodic Instability
Coefficient of Variation
*
I
Volume Instability
Coefficient of Variation
**
J
Temperature
Degrees (°C)
***
Pre-CNO Beginning
Pre-CNO Middle
Post-CNO End
Post-CNO 30 min

Caption: Supplemental Figure S4. Acute perturbation of *HoxA2-Cre* neurons in adult mice in a hypoxic protocol resulted in no respiratory changes under room air, increases in respiratory rate, tidal volume, minute ventilation, and $\dot{V}_E/\dot{V}_{O2}$ under hypoxic conditions, and a reduced body temperature 30 minutes after the end of the assay. Baseline room air respiratory parameters were measured pre-CNO, followed by CNO injection (10 mg/kg) and another exposure to room air and a single exposure to hypoxia (10% O_2). No pre-CNO 10% O_2 measurement was made to avoid potential confounds of hypoxic plasticity on the post-hypoxic response. After CNO administration, under room air conditions *HoxA2-Cre; P_hM4D* animals showed no significant change in all measured respiratory parameters as compared to pre-CNO values and sibling controls. Under post-CNO hypoxic conditions, *HoxA2-Cre; P_hM4D* animals had increased respiratory rate **(A)**, tidal volume **(B)**, minute ventilation **(C)**, and $\dot{V}_E/\dot{V}_{O2}$ **(E)** with no significant change in oxygen consumption **(D)**, apnea **(F)** and sigh frequency **(G)**, or periodic **(H)** and volume instability **(I)** as compared to sibling controls. *HoxA2-Cre; P_hM4D* animals also showed a significant reduction in temperature 30 minutes after the end of the respiratory assay as compared to sibling controls **(J).** Statistical significance was determined using a linear mixed-effects regression model with animal type (experimental or control) and CNO treatment (pre or post) as fixed effects and animal ID as a random effect. *$p<0.05$, **$p<0.01$, ***$p<0.001$.

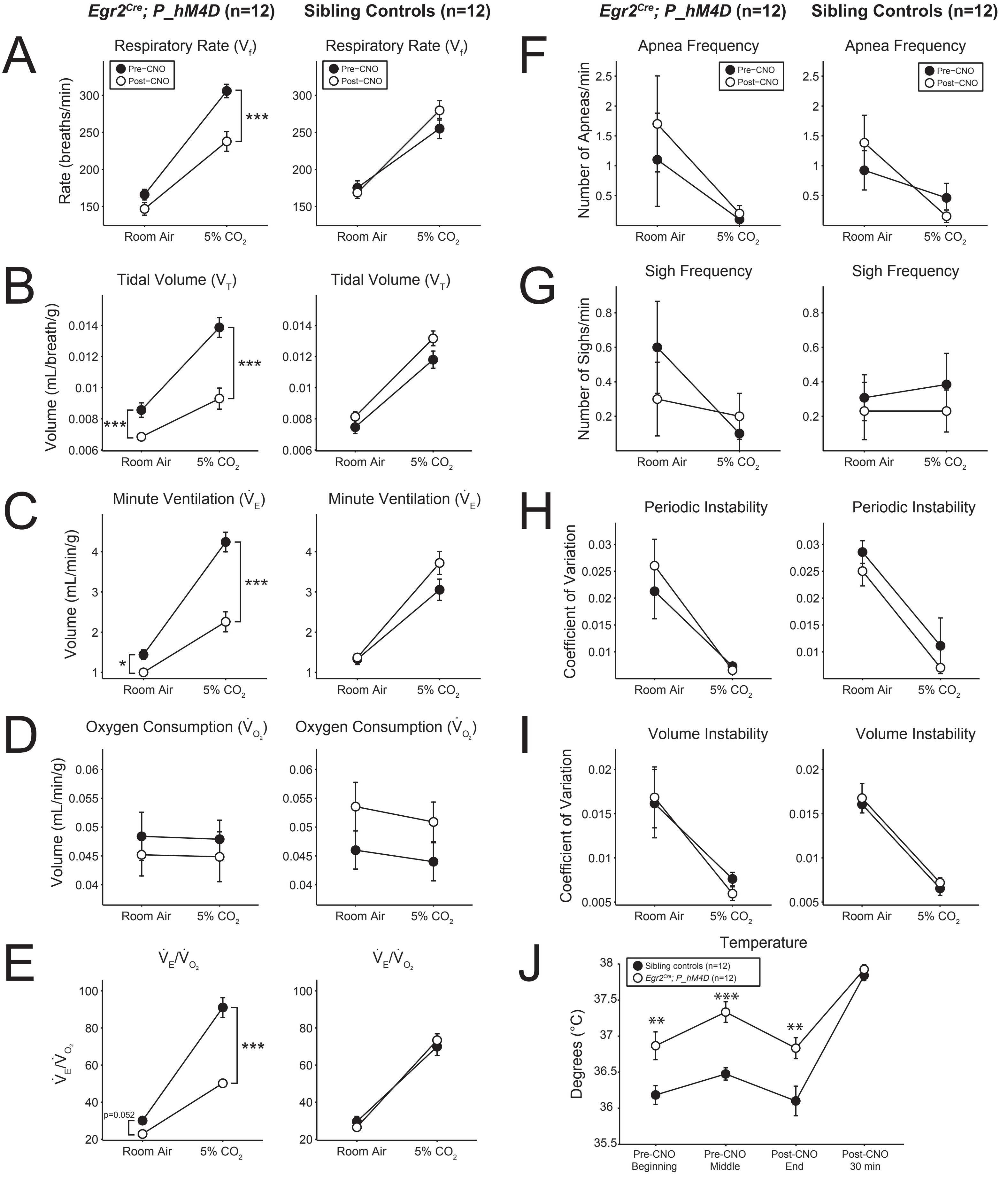
Egr2Cre; P_hM4D (n=12)
Sibling Controls (n=12)
Egr2Cre; P_hM4D (n=12)
Sibling Controls (n=12)
A
Respiratory Rate (Vf)
Pre−CNO
Post−CNO
Rate (breaths/min)
Room Air
5% CO2
***
B
Tidal Volume (VT)
Volume (mL/breath/g)
***
***
C
Minute Ventilation (V̇E)
Volume (mL/min/g)
*
***
D
Oxygen Consumption (V̇O2)
Volume (mL/min/g)
E
V̇E/V̇O2
p=0.052
***
F
Apnea Frequency
Number of Apneas/min
G
Sigh Frequency
Number of Sighs/min
H
Periodic Instability
Coefficient of Variation
I
Volume Instability
Coefficient of Variation
J
Temperature
Sibling controls (n=12)
Egr2Cre; P_hM4D (n=12)
Degrees (°C)
**
***
**
Pre-CNO Beginning
Pre-CNO Middle
Post-CNO End
Post-CNO 30 min

Caption: Supplemental Figure S5. Acute perturbation of *Egr2*^{Cre} neurons in adult mice in a hypercapnic protocol resulted in a reduction in respiratory rate, tidal volume, minute ventilation, and $\dot{V}_E/\dot{V}_{O2}$ under room air conditions, and a reduction in respiratory rate, tidal volume, minute ventilation and $\dot{V}_E/\dot{V}_{O2}$ under hypercapnic conditions. Baseline respiratory parameters were measured pre-CNO under room air and hypercapnic (5% CO_2) conditions, followed by CNO injection (10 mg/kg) and another exposure to room air and hypercapnia. After CNO administration, under both room air and hypercapnic conditions *Egr2*^{Cre}*; P_hM4D* animals showed a significant reduction in respiratory rate **(A)**, tidal volume **(B)**, and minute ventilation **(C)**, with no changes in oxygen consumption **(D)**, apnea **(F)** and sigh frequency **(G)**, or periodic **(H)** and volume instability **(I)** as compared to pre-CNO values and sibling controls. *Egr2Cre; P_hM4D* animals showed a trend toward reduced $\dot{V}_E/\dot{V}_{O2}$ **(E)** under room air conditions and had a reduced $\dot{V}_E/\dot{V}_{O2}$ under hypercapnic conditions. *Egr2*^{Cre}*; P_hM4D* animals also showed a significantly higher temperature pre-CNO in the beginning and middle and post-CNO at the end of the assay as compared to sibling controls **(J)**. Statistical significance was determined using a linear mixed-effects regression model with animal type (experimental or control) and CNO treatment (pre or post) as fixed effects and animal ID as a random effect. *$p<0.05$, **$p<0.01$, ***$p<0.001$.

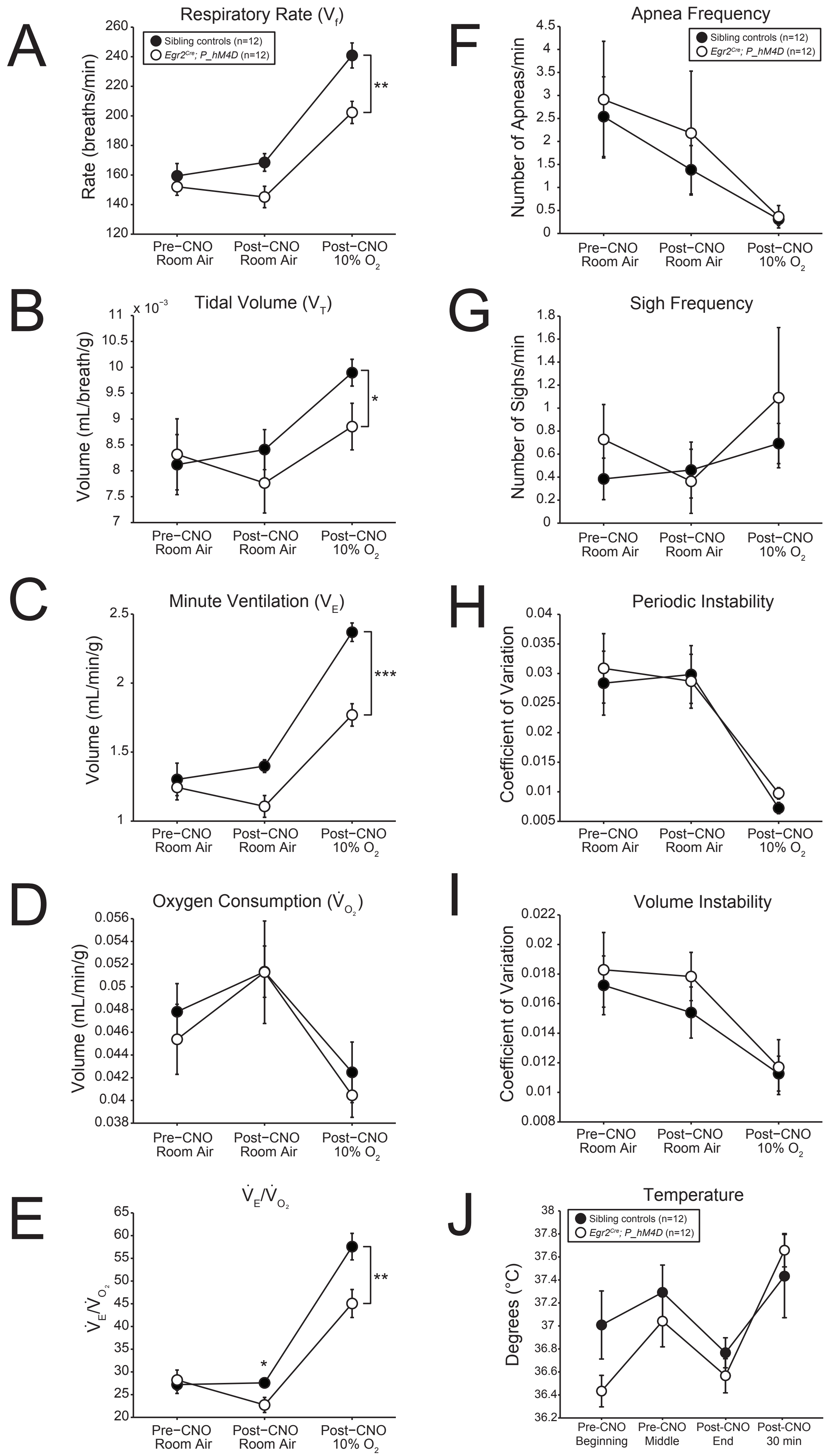

A
Respiratory Rate (V_f)
Rate (breaths/min)
Sibling controls (n=12)
Egr2Cre; P_hM4D (n=12)
**
B
Tidal Volume (V_T)
x 10−3
Volume (mL/breath/g)
*
C
Minute Ventilation (V_E)
Volume (mL/min/g)
***
D
Oxygen Consumption ($\dot{V}_{O_2}$)
Volume (mL/min/g)
E
$\dot{V}_E/\dot{V}_{O_2}$
*
**
Pre−CNO Room Air
Post−CNO Room Air
Post−CNO 10% O_2
F
Apnea Frequency
Number of Apneas/min
G
Sigh Frequency
Number of Sighs/min
H
Periodic Instability
Coefficient of Variation
I
Volume Instability
Coefficient of Variation
J
Temperature
Degrees (°C)
Pre-CNO Beginning
Pre-CNO Middle
Post-CNO End
Post-CNO 30 min

Caption: Supplemental Figure S6. Acute perturbation of *Egr2*^{Cre} neurons in adult mice in a hypoxic protocol resulted in a reduction in $\dot{V}_E/\dot{V}_{O2}$ under room air, and a reduction in respiratory rate, tidal volume, minute ventilation, and $\dot{V}_E/\dot{V}_{O2}$ under hypoxic conditions. Baseline room air respiratory parameters were measured pre-CNO, followed by CNO injection (10 mg/kg) and another exposure to room air and a single exposure to hypoxia (10% O_2). No pre-CNO 10% O_2 measurement was made to avoid potential confounds of hypoxic plasticity on the post-hypoxic response. After CNO administration, under room air conditions *Egr2*^{Cre}*; P_hM4D* animals showed a significant reduction in $\dot{V}_E/\dot{V}_{O2}$ **(E)** with no significant changes in respiratory rate **(A)**, tidal volume **(B)**, minute ventilation **(C)**, oxygen consumption **(D)**, apnea **(F)** and sigh frequency **(G)**, or periodic **(H)** and volume instability **(I)** as compared to pre-CNO values and sibling controls. Under post-CNO hypoxic conditions, *Egr2*^{Cre}*; P_hM4D* animals had a reduced respiratory rate **(A)**, tidal volume **(B)**, minute ventilation **(C)**, and $\dot{V}_E/\dot{V}_{O2}$ **(E)** with no significant change in other respiratory parameters as compared to sibling controls. No significant changes were seen in temperature **(J)**. Statistical significance was determined using a linear mixed-effects regression model with animal type (experimental or control) and CNO treatment (pre or post) as fixed effects and animal ID as a random effect. *p<0.05, **p<0.01, ***p<0.001.

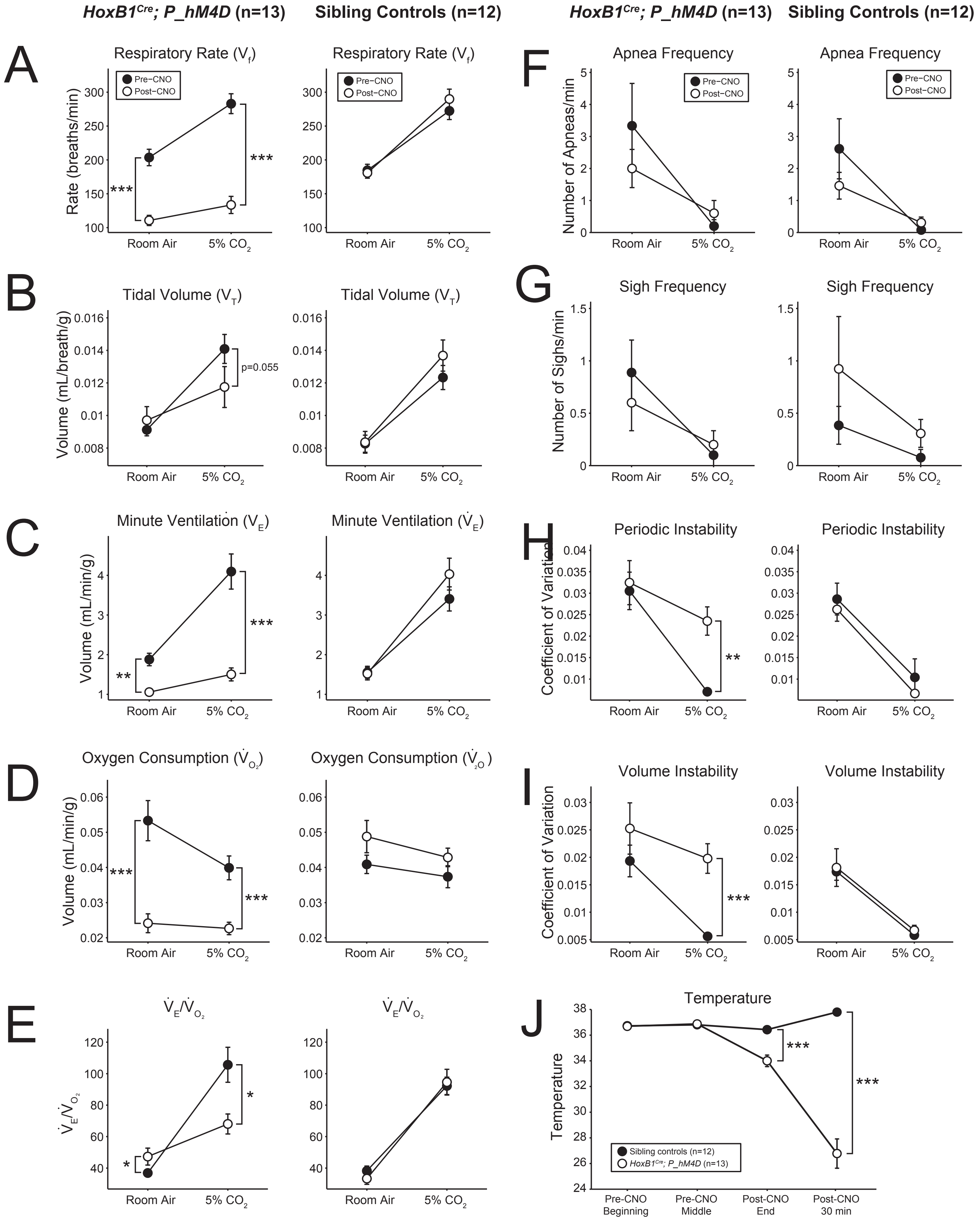

HoxB1^Cre; P_hM4D (n=13)
Sibling Controls (n=12)
A
Respiratory Rate (Vf)
Rate (breaths/min)
Pre-CNO
Post-CNO
***
Room Air
5% CO2
B
Tidal Volume (VT)
Volume (mL/breath/g)
p=0.055
C
Minute Ventilation (VE)
Volume (mL/min/g)
**
D
Oxygen Consumption (VO2)
E
VE/VO2
*
F
Apnea Frequency
Number of Apneas/min
G
Sigh Frequency
Number of Sighs/min
H
Periodic Instability
Coefficient of Variation
I
Volume Instability
J
Temperature
Sibling controls (n=12)
HoxB1^Cre; P_hM4D (n=13)
Pre-CNO Beginning
Pre-CNO Middle
Post-CNO End
Post-CNO 30 min

Caption: Supplemental Figure S7. Acute perturbation of *HoxB1^{Cre}* neurons in adult mice in a hypercapnic protocol resulted in a reduction in respiratory rate, minute ventilation, oxygen consumption and an increase in $\dot{V}_E/\dot{V}_{O2}$ under room air conditions; a reduction in respiratory rate, minute ventilation and $\dot{V}_E/\dot{V}_{O2}$ under hypercapnic conditions, and decreased temperature at the end of the assay and 30 minutes post. Baseline respiratory parameters were measured pre-CNO under room air and hypercapnic (5% CO_2) conditions, followed by CNO injection (10 mg/kg) and another exposure to room air and hypercapnia. After CNO administration, under room air *HoxB1^{Cre}; P_hM4D* animals showed a significant reduction in respiratory rate **(A)**, minute ventilation **(C)**, oxygen consumption **(D)** resulting in a significantly increased $\dot{V}_E/\dot{V}_{O2}$ **(E)** with no changes in tidal volume **(B),** apnea **(F)** and sigh frequency **(G)**, or periodic **(H)** and volume instability **(I)** as compared to pre-CNO values and sibling controls. Under hypercapnic conditions, *HoxB1^{Cre}; P_hM4D* animals showed significantly reduced respiratory rate **(A),** minute ventilation **(C)**, oxygen consumption **(D)**, and $\dot{V}_E/\dot{V}_{O2}$ **(E)** and increased periodic **(H)** and volume instability **(I)** as compared to pre-CNO values and sibling controls. *HoxB1^{Cre}; P_hM4D* animals also showed a reduced temperature immediately at the end of the assay and 30 minutes after the end **(J)**. Statistical significance was determined using a linear mixed-effects regression model with animal type (experimental or control) and CNO treatment (pre or post) as fixed effects and animal ID as a random effect. *p<0.05, **p<0.01, ***p<0.001.

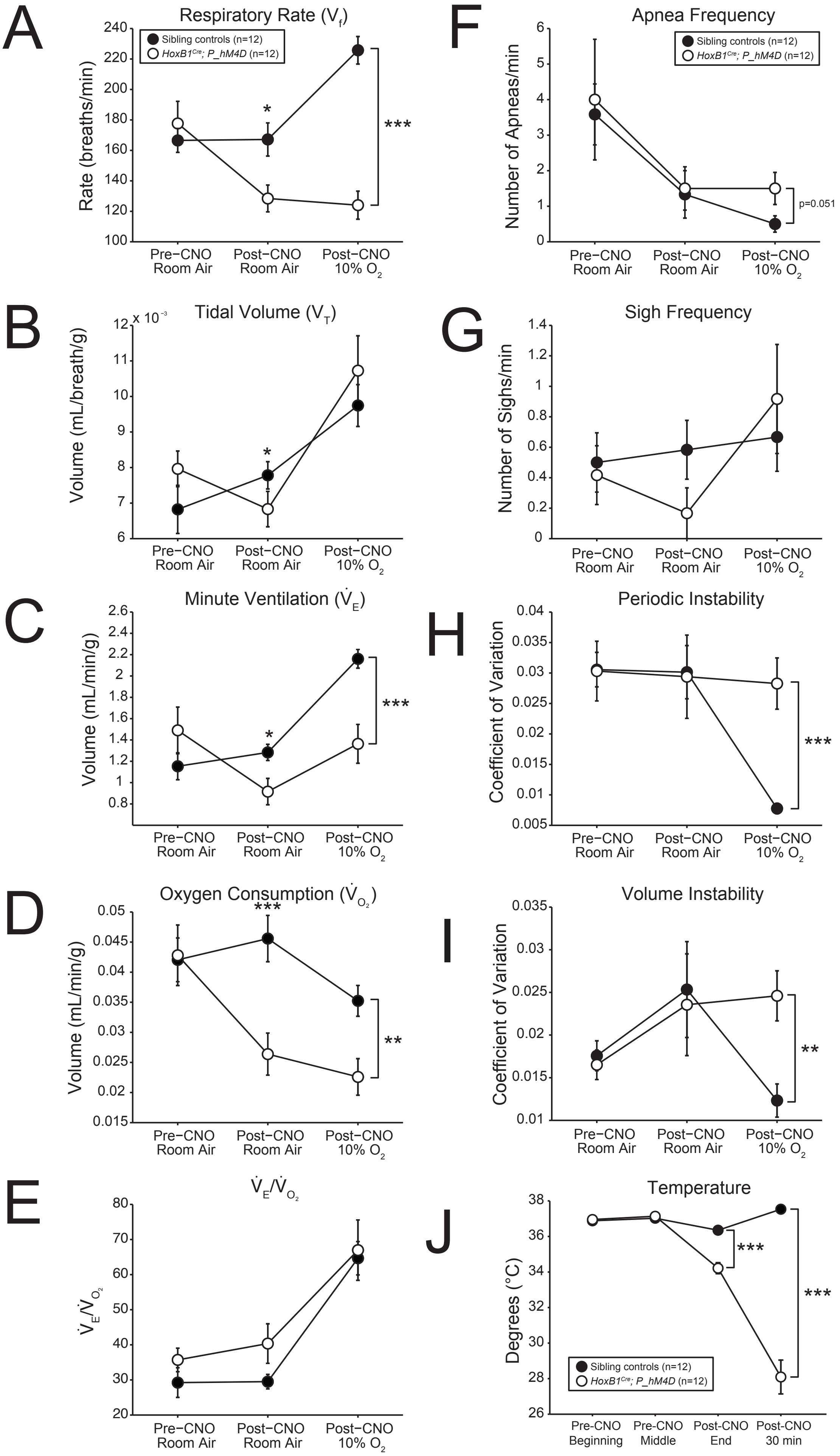
A
Respiratory Rate (V_f)
Sibling controls (n=12)
$HoxB1^{Cre}$; P_hM4D (n=12)
Rate (breaths/min)
*
***
Pre−CNO Room Air
Post−CNO Room Air
Post−CNO 10% O_2
B
Tidal Volume (V_T)
x 10^{-3}
Volume (mL/breath/g)
*
C
Minute Ventilation ($\dot{V}_E$)
Volume (mL/min/g)
*
***
D
Oxygen Consumption ($\dot{V}_{O_2}$)
Volume (mL/min/g)
***
**
E
$\dot{V}_E/\dot{V}_{O_2}$
F
Apnea Frequency
Number of Apneas/min
p=0.051
G
Sigh Frequency
Number of Sighs/min
H
Periodic Instability
Coefficient of Variation
***
I
Volume Instability
Coefficient of Variation
**
J
Temperature
Degrees (°C)
***
***
Pre-CNO Beginning
Pre-CNO Middle
Post-CNO End
Post-CNO 30 min

Caption: Supplemental Figure S8. Acute perturbation of *HoxB1^Cre^* neurons in adult mice in a hypoxic protocol resulted in a reduction in respiratory rate, tidal volume, minute ventilation, and oxygen consumption under room air, reduced respiratory rate, minute ventilation, and oxygen consumption, under hypoxic conditions, and lower temperature immediately at the end of the assay and post 30 minutes. Baseline room air respiratory parameters were measured pre-CNO, followed by CNO injection (10 mg/kg) and another exposure to room air and a single exposure to hypoxia (10% O_2). No pre-CNO 10% O_2 measurement was made to avoid potential confounds of hypoxic plasticity on the post-hypoxic response. After CNO administration, under room air conditions *HoxB1^Cre^; P_hM4D* animals showed a significant reduction in respiratory rate **(A),** tidal volume **(B)**, minute ventilation **(C)**, and oxygen consumption **(D)** with no significant changes in $\dot{V}_E/\dot{V}_{O2}$ **(E),** apnea **(F)** and sigh frequency **(G)**, or periodic **(H)** and volume instability **(I)** as compared to pre-CNO values and sibling controls. Under post-CNO hypoxic conditions, *HoxB1^Cre^; P_hM4D* animals had a reduced respiratory rate **(A)**, minute ventilation **(C)**, and oxygen consumption **(D)** and increased periodic **(H)** and volume instability **(I)** as compared to sibling controls. *HoxB1^Cre^; P_hM4D* animals also showed a reduced temperature immediately at the end of the assay and 30 minutes after the end **(J)**. Statistical significance was determined using a linear mixed-effects regression model with animal type (experimental or control) and CNO treatment (pre or post) as fixed effects and animal ID as a random effect. *p<0.05, **p<0.01, ***p<0.001.

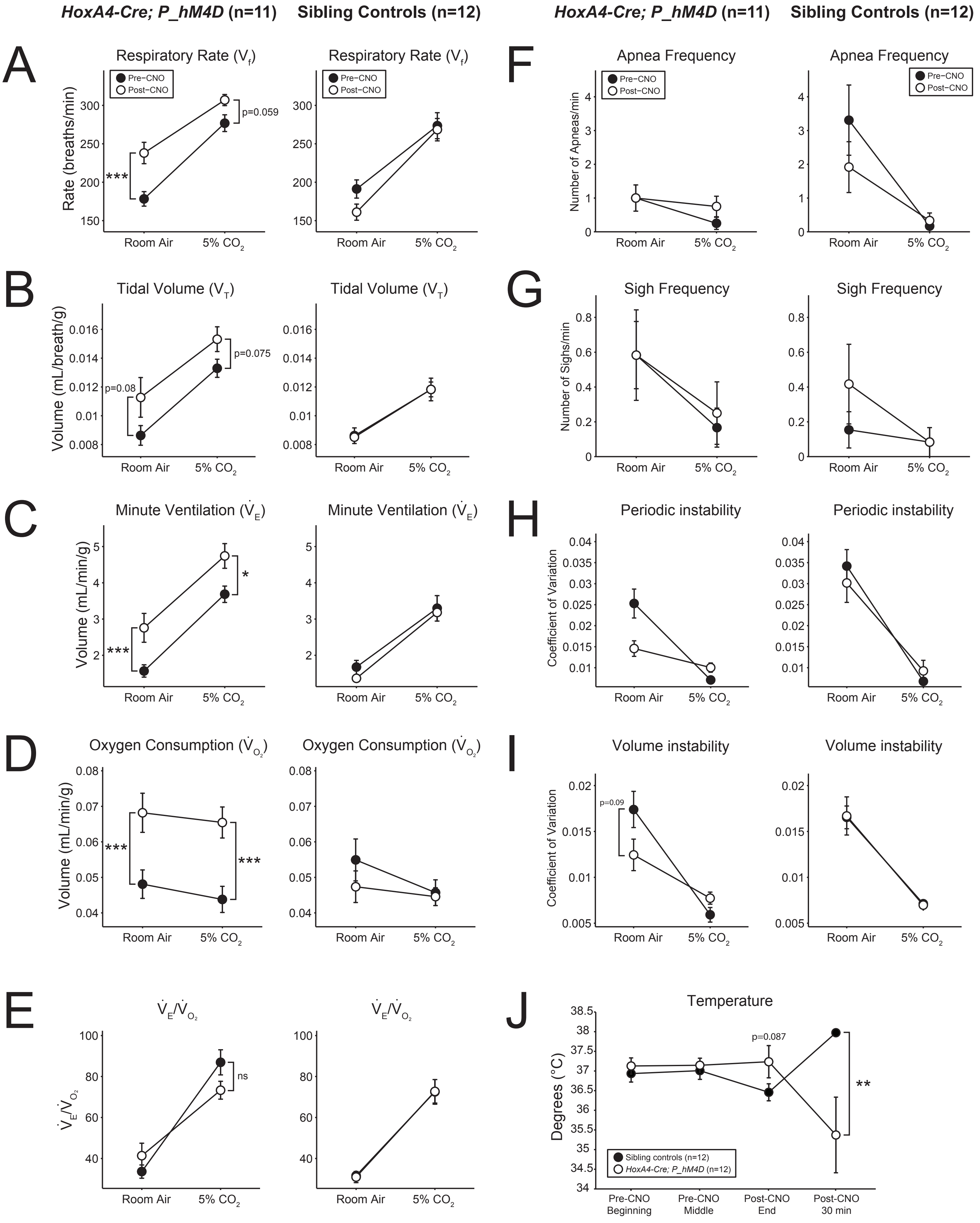
HoxA4-Cre; P_hM4D (n=11)
Sibling Controls (n=12)
HoxA4-Cre; P_hM4D (n=11)
Sibling Controls (n=12)
A
Respiratory Rate (Vf)
Pre-CNO
Post-CNO
Rate (breaths/min)
***
p=0.059
Room Air
5% CO2
B
Tidal Volume (VT)
Volume (mL/breath/g)
p=0.08
p=0.075
C
Minute Ventilation (V̇E)
Volume (mL/min/g)
***
*
D
Oxygen Consumption (V̇O2)
Volume (mL/min/g)
***
***
E
V̇E/V̇O2
ns
F
Apnea Frequency
Number of Apneas/min
G
Sigh Frequency
Number of Sighs/min
H
Periodic instability
Coefficient of Variation
I
Volume instability
p=0.09
J
Temperature
Degrees (°C)
p=0.087
**
Sibling controls (n=12)
HoxA4-Cre; P_hM4D (n=12)
Pre-CNO Beginning
Pre-CNO Middle
Post-CNO End
Post-CNO 30 min

Caption: Supplemental Figure S9. Acute perturbation of *HoxA4-Cre* neurons in adult mice in a hypercapnic protocol resulted in increased respiratory rate, minute ventilation, and oxygen consumption under room air; increased minute ventilation and oxygen consumption under hypercapnic conditions; and a reduced temperature 30 minutes after the end of the assay. Baseline respiratory parameters were measured pre-CNO under room air and hypercapnic (5% CO_2) conditions, followed by CNO injection (10 mg/kg) and another exposure to room air and hypercapnia. After CNO administration, under room air conditions, *HoxA4-Cre; P_hM4D* animals showed a significant increase in respiratory rate **(A)**, minute ventilation **(C)**, and oxygen consumption **(D)** with no changes in tidal volume **(B)**, $\dot{V}_E/\dot{V}_{O2}$ **(E)**, apnea **(F)** and sigh frequency **(G)**, or periodic **(H)** and volume instability **(I)** as compared to pre-CNO values and sibling controls. Under hypercapnic conditions, *HoxA4-Cre; P_hM4D* animals showed a significant increase in minute ventilation and oxygen consumption with no changes in other respiratory parameters. *HoxA4-Cre; P_hM4D* animals also showed a reduced temperature 30 minutes after the end of the assay **(J)**. Statistical significance was determined using a linear mixed-effects regression model with animal type (experimental or control) and CNO treatment (pre or post) as fixed effects and animal ID as a random effect. *p<0.05, **p<0.01, ***p<0.001.

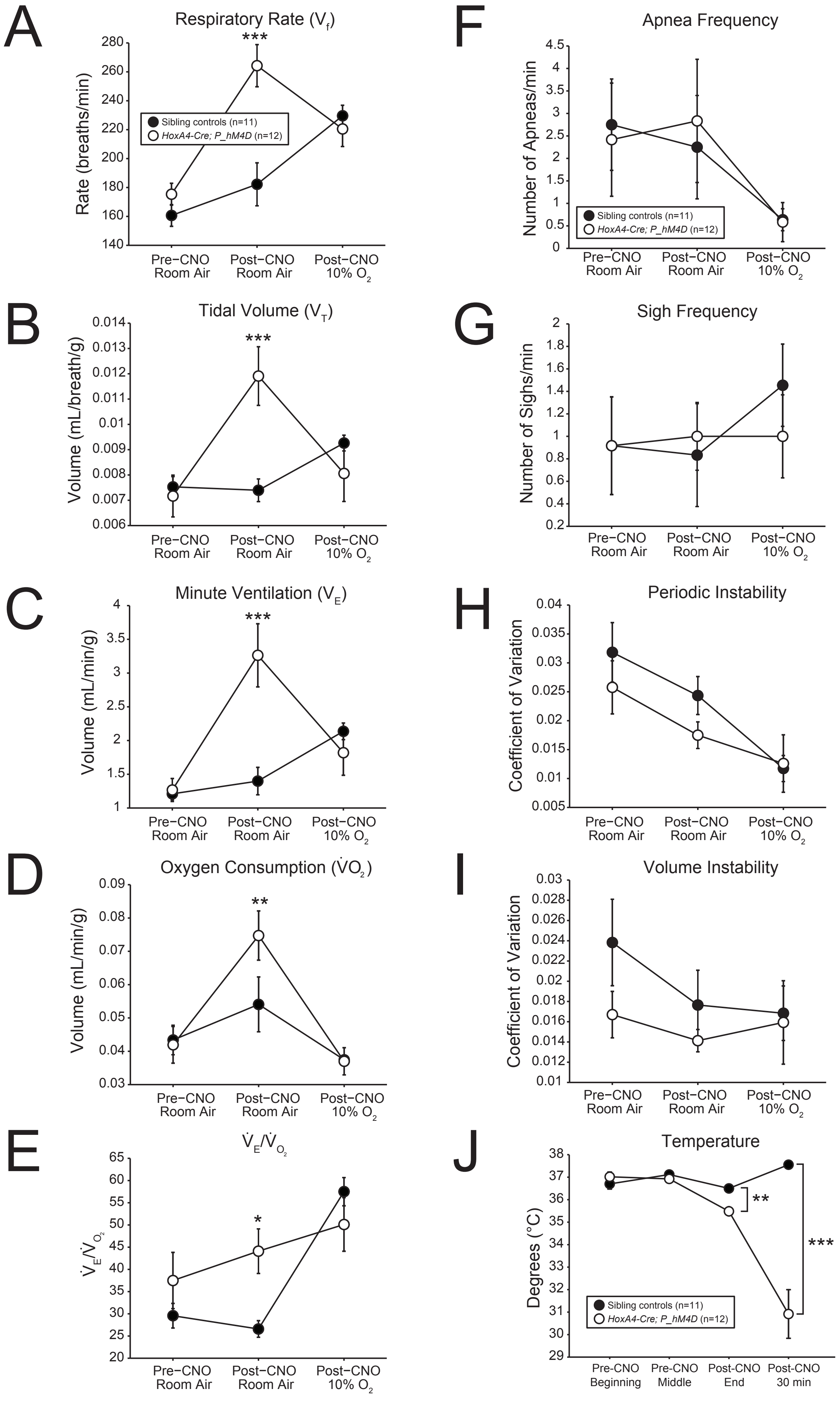

A
Respiratory Rate (V_f)
Rate (breaths/min)
***
Sibling controls (n=11)
HoxA4-Cre; P_hM4D (n=12)
Pre−CNO Room Air
Post−CNO Room Air
Post−CNO 10% O_2
B
Tidal Volume (V_T)
Volume (mL/breath/g)
***
C
Minute Ventilation (V_E)
Volume (mL/min/g)
***
D
Oxygen Consumption ($\dot{V}O_2$)
Volume (mL/min/g)
**
E
$\dot{V}_E/\dot{V}_{O_2}$
*
F
Apnea Frequency
Number of Apneas/min
G
Sigh Frequency
Number of Sighs/min
H
Periodic Instability
Coefficient of Variation
I
Volume Instability
Coefficient of Variation
J
Temperature
Degrees (°C)
**
***
Pre-CNO Beginning
Pre-CNO Middle
Post-CNO End
Post-CNO 30 min

Caption: Supplemental Figure S10. Acute perturbation of *HoxA4-Cre* neurons in adult mice in a hypoxic protocol resulted in increased respiratory rate, tidal volume, minute ventilation, oxygen consumption, and $\dot{V}_E/\dot{V}_{O2}$ under both room air conditions; no change under hypoxic conditions, and a reduced temperature immediately following and 30 minutes after the end of the assay. Baseline room air respiratory parameters were measured pre-CNO, followed by CNO injection (10 mg/kg) and another exposure to room air and a single exposure to hypoxia (10% O_2). No pre-CNO 10% O_2 measurement was made to avoid potential confounds of hypoxic plasticity on the post-hypoxic response. After CNO administration, under room air, *HoxA4-Cre; P_hM4D* animals showed a significant increase in respiratory rate **(A)**, tidal volume **(B)**, minute ventilation **(C)**, oxygen consumption **(D)** and $\dot{V}_E/\dot{V}_{O2}$ with no changes in apnea **(F)** and sigh frequency **(G)**, or periodic **(H)** and volume instability **(I)** as compared to pre-CNO values and sibling controls. *HoxA4-Cre; P_hM4D* animals also showed a reduced temperature immediately after the end of the assay and 30 minutes after the end of the assay **(J)**. Statistical significance was determined using a linear mixed-effects regression model with animal type (experimental or control) and CNO treatment (pre or post) as fixed effects and animal ID as a random effect. *p<0.05, **p<0.01, ***p<0.001.
